# CBPtools: a Python package for regional connectivity-based parcellation

**DOI:** 10.1007/s00429-020-02046-1

**Published:** 2020-03-06

**Authors:** Niels Reuter, Sarah Genon, Shahrzad Kharabian Masouleh, Felix Hoffstaedter, Xiaojin Liu, Tobias Kalenscher, Simon B. Eickhoff, Kaustubh R. Patil

**Affiliations:** 1 Institute of Systems Neuroscience, Heinrich-Heine University, Düsseldorf, Germany; 2 Institute of Neuroscience and Medicine, Brain and Behaviour (INM-7), Research Centre Jülich, Jülich, Germany; 3 Comparative Psychology, Institute of Experimental Psychology, Heinrich-Heine University Düsseldorf, Düsseldorf, Germany

**Keywords:** Connectivity-based parcellation, Clustering, Resting state, Diffusion-weighted imaging, Software

## Abstract

Regional connectivity-based parcellation (rCBP) is a widely used procedure for investigating the structural and functional differentiation within a region of interest (ROI) based on its long-range connectivity. No standardized software or guidelines currently exist for applying rCBP, making the method only accessible to those who develop their own tools. As such, there exists a discrepancy between the laboratories applying the procedure each with their own software solutions, making it difficult to compare and interpret the results. Here, we outline an rCBP procedure accompanied by an open source software package called *CBPtools*. *CBPtools* is a Python (version 3.5+) package that allows users to run an extensively evaluated rCBP analysis workflow on a given ROI. It currently supports two modalities: resting-state functional connectivity and structural connectivity based on diffusion-weighted imaging, along with support for custom connectivity matrices. Analysis parameters are customizable and the workflow can be scaled to a large number of subjects using a parallel processing environment. Parcellation results with corresponding validity metrics are provided as textual and graphical output. Thus, *CBPtools* provides a simple plug-and-play, yet customizable way to conduct rCBP analyses. By providing an open-source software we hope to promote reproducible and comparable rCBP analyses and, importantly, make the rCBP procedure readily available. Here, we demonstrate the utility of *CBPtools* using a voluminous data set on an average compute-cluster infrastructure by performing rCBP on three ROIs prominently featured in parcellation literature.

**Electronic supplementary material:**

The online version of this article (10.1007/s00429-020-02046-1) contains supplementary material, which is available to authorized users.

## Introduction

Mapping the human brain in an effort to understand its organizational principles is a monumental task, dating back to the early 1900s with Korbinian Brodmann’s famous publication on ’Vergleichende Lokalisationslehre der Großhirnrinde’ (Localization in the cerebral cortex; Brodmann (1909)). The advent of modern non-invasive in vivo neuroimaging technologies, such as magnetic resonance imaging (MRI), has been driving growth in this research field. Accompanying progress in neuroimaging data analysis techniques allow a range of connectivity measurements from various MRI modalities. Brain organization can then be probed by analyzing the patterns in these measurements. One such technique is connectivity-based parcellation (CBP), an umbrella term for a widely used and diverse set of procedures to delineate whole- and regional brain organization, originally conceived by Behrens et al. (2003) in their seminal work on the thalamus.

A common approach to mapping the human brain through CBP is to cluster voxels/vertices into parcels. Here, we focus on regional CBP (rCBP). A clustering algorithm is used to group voxels/vertices within a given region of interest (ROI) based on similarity in their connection strengths to a set of target voxels/vertices, i.e., their connectivity profile. Voxels clustered together form homogeneous units, i.e., parcels, with regard to the measured connectivity marker that best describes the input data at hand. The parcels are often spatially consistent, as neighboring voxels usually exhibit more similar connectivity patterns than those further away. Thus, the rCBP procedure can map functional or structural subdivisions/clusters within a particular ROI. rCBP derived parcels are known to match with histological parcellation (Bzdok et al. 2013), but they may also provide subdivisions pertaining to different sources of information not revealed by cytoarchitectonic mapping alone (Clos et al. 2013). As each MRI modality yields a different aspect of brain connectivity, rCBP on each modality can yield differing parcellations with different interpretations. Commonly used imaging modalities include, but are not limited to, resting-state blood oxygen level-dependent (BOLD) time series used to measure task-independent functional connectivity and diffusion-weighted imaging (DWI)-based probabilistic diffusion tractography to estimate anatomical fiber connectivity, as well as meta-analytic connectivity modeling (MACM) as a measure of task-dependent functional connectivity and co-activation patterns. Due to the different interpretations that may result from each modality, a multimodal approach [e.g., Genon et al. (2018) and Plachti et al. (2019)] may be used to compare unimodal parcellations.

Various methods can be employed at different steps within the rCBP procedure. For instance, unlike whole-brain parcellations (Schaefer et al. 2018), rCBP focuses on a particular ROI, hence allowing an in-depth analysis by uncovering the internal differentiation of a region. Our approach relies on using static rather than dynamic (Hutchison et al. 2013; Ji et al. 2016) patterns of connectivity. Moreover, we use a hard cluster assignment employing an unsupervised machine learning algorithm (*k*-means, spectral, or agglomerative clustering) as opposed to probabilistic, graded (Bajada et al. 2017), or boundary mapping (Cohen et al. 2008) approaches. For a more detailed overview of the rCBP procedure, we recommend reading Eickhoff et al. (2015) and Eickhoff et al. (2018).

Despite its popularity, rCBP is challenging and time consuming to employ without the necessary tools. As neuroscience makes a transition toward big data, with prominent examples such as the Human Connectome Project (HCP) (Van Essen et al. 2013) and the 1000BRAINS study (Caspers et al. 2014) having well over 1000 subjects, it becomes an increasing necessity to add support for high-throughput computation and parallel processing. Furthermore, the numerous options available at each step of the rCBP procedure paired with the absence of uniform guidelines make it difficult to have comparable results. For example, the choice of clustering algorithm may influence the clustering results, with options such as *k*-means clustering, spectral clustering, or hierarchical clustering (Hastie et al. 2013; Von Luxburg 2007).

To resolve these issues, we introduce *CBPtools*, an open-source distributed workflow for rCBP enclosed in a Python package. By unifying the methodological choices behind the procedure into a customizable workflow, we offer a fast, stable, and reproducible means to parcellate the brain regions. Furthermore, computational demands highlighted by complex algorithms and large data sets are mitigated by efficient parallel execution of the procedure. *CBPtools* offers a common working ground to effortlessly and efficiently conduct reproducible and data-driven parcellation analyses.

**Fig. 1:**
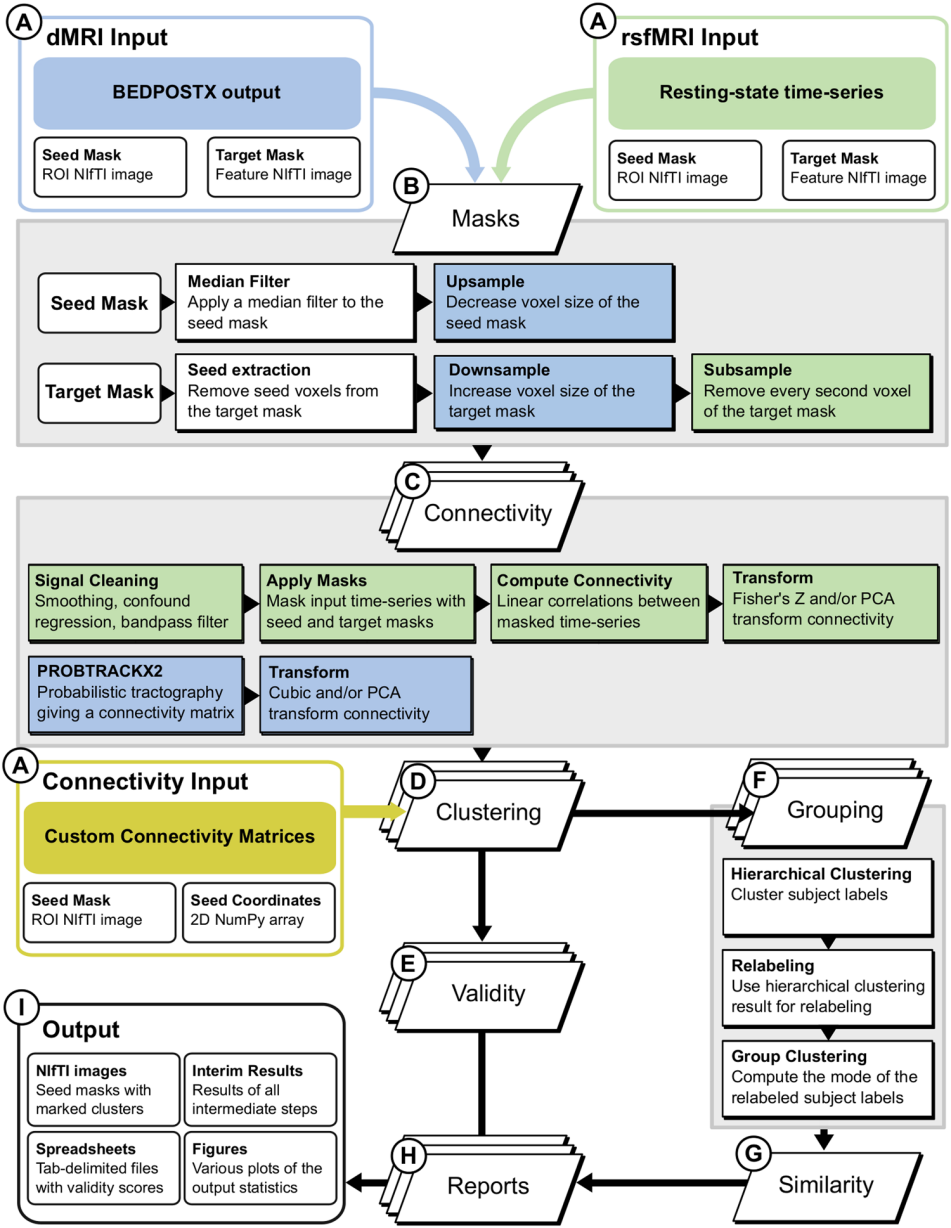
*CBPtools* workflow for applying the rCBP procedure to diffusion MRI (dMRI) or resting-state functional MRI (rsfMRI) data. After customizing the parameters of the procedure, input data (A) is processed through each step (B through H) of the workflow. Note that the different types of inputs are not processed in parallel, but must instead be set up and executed as different *CBPtools* projects. Steps are executed in parallel whenever possible (e.g., the connectivity, clustering, validity, grouping, and reports steps). The mask preprocessing and connectivity steps use different methods based on the input modality (i.e., dMRI or rsfMRI, highlighted in blue and green, respectively). Thus, methods with a green background are only performed on rsfMRI data, and methods with a blue background are only performed on dMRI data. Alternatively custom connectivity matrices can be given as input, which will skip steps B and C

## Materials and methods

### CBPtools overview

*CBPtools* parcellates an ROI and provides the output as NIfTI images along with commonly used cluster-validity metrics. The tool’s approach and methods are derived from a substantial body of parcellation works (Wang et al. 2015; Bzdok et al. 2015; Chase et al. 2015; Barron et al. 2015; Hardwick et al. 2015; Eickhoff et al. 2016; Muhle-Karbe et al. 2016; Genon et al. 2017, 2018; Plachti et al. 2019) and consist of a customizable rCBP workflow allowing users to specify the input data and a range of parameters through a configuration file. *CBPtools* can calculate connectivity matrices from resting-state or DWI data, but they may instead be provided directly as input. It then computes parcellations based on the connectivity matrices (projected onto NIfTI images of the ROI and as NumPy array files, as well as 3D voxel plots) and outputs validity metrics for their evaluation. Note that the procedure outlined here utilizes hard clustering. Therefore, when connectivity markers are assumed to change through soft transition (i.e., showing a gradient), parcels generated through this procedure should not be interpreted as neurobiological units, but as a simplified data representation (or compression model). Figure 1 provides an overview of the workflow procedure, with each step detailed in the following sections.

To illustrate both the usage of *CBPtools* and the output it provides, the resting-state and DWI modalities of the HCP data (Van Essen et al. 2013) were used to parcellate three regions that have been frequently analyzed using the rCBP procedure: the right (R) Insula, R amygdala, and an ROI comprising R presupplementary motor area (preSMA) and R supplementary motor area (SMA) (see "Example data" for details).

#### Architecture

*CBPtools* is written in Python (version 3.5+) to exploit Python’s prolific presence in the data science community and can be installed with pip (‘*pip install cbptools*’). We capitalized on pre-existing and widely used packages, such as NumPy, SciPy, NiBabel, and scikit-learn, which provide a range of methods needed for the rCBP procedure. This makes the software very accessible on account of Python and its libraries being free and open source, as well as compatible with many operating systems.

*CBPtools* makes use of snakemake (Köster and Rahmann 2012), an easy-to-use and well documented workflow management system with parallel processing capabilities that allows the workflow execution to be scaled to various processing environments (i.e., server, cluster, grid, or cloud environments). Through snakemake, *CBPtools* is compatible with job schedulers that support shell script (such as SLURM and qsub). Furthermore, *CBPtools* can be resumed with partially processed data (e.g., due to hardware failure) making it stable and efficient for use on real world data. The combination of snakemake’s command line execution and an easily modifiable configuration file make it possible to set up and run the software without any programming knowledge.

For a more detailed instruction on the use of the package, please visit the repository at https://github.com/inm7/cbptools or our online documentation (Reuter 2019).

#### Setup and input specification

Processing parameters and options can be defined by means of a configuration file, for which the parameter choices and fields will be validated and logged. Errors during the setup must first be resolved before proceeding, but warnings are not critical to the execution of *CBPtools*. However, they may give rise to unexpected results and should not be ignored. Upon completion of the setup, a new project folder is created at a user-specified location, containing all files necessary to initiate the workflow. The *CBPtools* online documentation (Reuter 2019) has a more detailed overview of the setup process. We have also provided a quick-start guide in the Online Resource (Sect. 1.2 Usage example) as well as on the GitHub project page.

The input data are separated into modality-independent and modality-dependent categories. Modality-independent input data include (1) a binary three-dimensional NIfTI ROI file in the three-dimensional NIfTI image data format, (2) an optional three-dimensional target mask in the same data format, used to define the connections that are considered for each ROI voxel. If not provided by the user, the FSL (http://www.fmrib.ox.ac.uk/fsl/) distributed average Montreal Neurological Institute (MNI) 152 T1 whole-brain gray matter group template (2 mm isotropic) will be used as the target, in which case the required input data should match the same MNI152 template as well, and (3) a participants file as a tab-separated text file with a column called ‘*participant_id*’ containing all unique identifiers of the subjects to be included in the study.

Modality-dependent data depend on the selected input modality, i.e., rsfMRI, dMRI, or connectivity. For rsfMRI data, a 4D time series NIfTI image per subject must be provided, optionally accompanied by a tab-separated text file containing confounds for each time point as columns. *CBPtools* assumes that the rsfMRI data have been treated with necessary fMRI preprocessing including realignment and normalization to a template space. If the default target mask is used, then the template space must be MNI152 with 2 mm isotropic voxels. *CBPtools* also supports using native masks, but that prohibits the use of the default mask (for more information, see Online Resource Sect. 1.3.3 Single-subject parcellation). Denoising based on independent component analysis like Automatic Removal of Motion Artifacts (ICA-AROMA) (Pruim et al. 2015) or FMRIB’s ICA-based X-noiseifier (FIX) (Salimi-Khorshidi et al. 2014) is encouraged if suitable. In particular, FIX in combination with mean white matter and cerebrospinal fluid signal regression has been shown to work well in the context of rCBP (i.e., improved cluster stability and consistency of clusters between neuroimaging modalities) (Plachti et al. 2019). The dMRI modality requires input necessary to perform FSL’s probabilistic diffusion tractography (PROBTRACKX2), consisting of: (1) outputs from Bayesian estimation of diffusion parameters obtained using sampling techniques (BEDPOSTX), (2) a brain extraction (BET) binary mask file, (3) a transform file taking seed space to DTI space (either a FLIR matrix or FNIR warpfield; optional), and (4) a file describing the transformation from DTI space to seed space (optional unless input file 3 is defined). Each of these files is subject specific and can be obtained from FSL’s BEDPOSTX output. Connectivity matrices may be provided as source input in lieu of rsfMRI or dMRI data. They must be provided in a ROI-voxel by target-voxel shape, along with a binary three-dimensional mask of the ROI in NIfTI image data format, and a NumPy array of voxel coordinates in the order that the ROI voxels are represented in the connectivity matrix.

To define input data for the rCBP procedure, the full file paths must be added to the configuration file. *CBPtools* offers example configuration files using the ‘*cbptools example–get data-type*’ command, where data type is replaced by either ’connectivity’, ’rsfmri’, or ’dmri’, reflecting the different input data types. The absolute file path for subject-wise files should be specified as a template, i.e., containing the string *{participant_id}* which will be replaced by the ids of the subjects included in the rCBP project (through the inclusion of the aforementioned participant’s file). All input data should be quality controlled prior to using *CBPtools*, as only marginal validation is performed on the input data by *CBPtools*. Faulty data may halt processing until the issues are resolved, but in the worst case such data may provide output without explicit warnings that this output should not be trusted. Further specified during the setup are parameters to transform the connectivity matrices (e.g., cubic or Fisher’s *Z* transform, or feature reduction through principal component analysis), the clustering parameters (e.g., the range of *k* clusters requested) and validity measures, as well as the desired output file formats. Each of these parameters are likewise specified in the configuration file.

#### ROI mask preprocessing

There are various atlases and tools that can be used to respectively define an ROI and extract it as a binary mask. For instance, the JuBrain Anatomy Toolbox (Eickhoff et al. 2005) can be utilized to extract an ROI using probabilistic cytoarchitectonic maps. Alternatively, the FSL distributed atlases (e.g., the Harvard-Oxford Atlas) can likewise be used to extract an ROI. The mask must be a three-dimensional binary NIfTI image.

The ROI mask is validated for binarity and conformity to either an optionally provided target mask, or by default the group MNI template space (2 mm isotropic voxels, $91 \times 109 \times 91$ shape, and origin at *x* = 90, *y* = $-\,126$, *z* = $-\,72$). When connectivity matrices are given as input in lieu of rsfMRI or dMRI data, the ROI mask is only validated for binarity. For all other cases, it is important that input data are in the same space as the mask. It is possible to use a different space, but then a target mask must be provided in the desired space, which then both the ROI mask and input data must conform to. Without a user-provided target mask, all whole-brain gray matter voxels are used as target and subsampled (see below) by default (although this option can be turned off in the configuration file).

Available preprocessing steps are modality specific and all are optional. For the rsfMRI modality, the ROI mask can be median filtered and the target mask can be subsampled and have all ROI voxels removed from it. Median filtering replaces each voxel with the median of its neighboring voxels. For binarized images, this will remove voxels with too few neighbors and add voxels to the mask when they have many neighbors. It can be particularly useful for hand-drawn ROIs, as it removes sharp borders or stray voxels that would not naturally occur in most ROIs. Subsampling the target mask is recommended when smoothed BOLD time series are used. This means that only every second voxel in each dimension is kept under the spatial-smoothness assumption that neighboring voxels provide a relatively similar signal. This can significantly reduce computation time while preserving most of the information due to spatial smoothness. By choosing to remove all ROI voxels from the target mask, the ROI to ROI (i.e., within-ROI) connectivity is ignored. Within-ROI connectivity (i.e., connectivity between every pair of voxels within the ROI) tends to be high due to their relative proximity to one another and may therefore dominate the clustering. Whether doing so leads to better or more biologically relevant parcellation results, however, is unclear. Its application can also optionally remove a border around the ROI to reduce the influence of smoothing. For dMRI, in addition to median filtering, the ROI can also be upsampled. This upsampling option spreads the ROI voxels to cover a larger area (reflecting a higher resolution for use with PROBTRACKX2), while maintaining the same number of voxels (which is necessary so that ROI voxels can be mapped back upon the original ROI mask). Thus, voxels within the upsampled ROI will be spread out equidistantly over a larger area with no direct neighboring voxels as a result of not increasing their amount. The target mask can be downsampled from a higher to a lower resolution, resulting in fewer voxels covering the same space (i.e., larger voxels) which can reduce computation time for PROBTRACKX2.

#### Connectivity computation

To derive rsfMRI connectivity, the BOLD time series are optionally smoothed [using NiBabel’s (Brett et al. 2019) *nibabel.processing.smooth_image*], nuisance signal regressed (linear regression of confound time points on subject time series), and band-pass-filtered. An ROI-to-target connectivity matrix is calculated per subject using linear correlations between the ROI and target BOLD time series. A user can optionally choose for the connectivity matrices to be Fisher’s *Z* transformed and/or be subjected to linear dimensionality reduction (through principal component analysis). For dMRI, the PROBTRACKX2 output, a sparse ROI- by target-voxel connectivity matrix (*omatrix2*) per subject, is densified and cubic transformed, and can optionally be subjected to linear dimensionality reduction as well.

#### Individual- and group-level clustering

Connectivity from the previous step is given as input to the *k*-means algorithm [using scikit-learn’s (Pedregosa et al. 2011) *sklearn.cluster.KMeans*], separately for each subject and for each requested number of clusters *k*. Alternatively, agglomerative/hierarchical (Hastie et al. 2013) or spectral clustering (Von Luxburg 2007) can be used instead of *k*-means. The *k*-means algorithm was chosen as the default algorithm due to its popularity in CBP literature. The clustering algorithm assigns each ROI voxel/vertex to a cluster, effectively grouping similar voxels based on their connectivity profiles (for *k*-means this is done by minimizing the squared Euclidean distance between voxel features, i.e., the connectivity profiles and that of cluster centers). Thus, for each individual subject and a given modality, the output is the parcellation of the ROI voxels/vertices.

As the individual-level cluster ids are arbitrary, to determine a group parcellation that best describes all included subjects first, the individual clusterings at each *k* are relabeled such that similar clusters get assigned the same cluster ids across subjects. For each *k*, all individual clusterings are given as input to SciPy’s (Jones et al. 2001) implementation of hierarchical clustering (*scipy.cluster.hierarchy*) using the Hamming distance metric and a user-defined linkage algorithm (defaults to ’complete’). Hamming distance is used to take the arbitrary nature of the cluster ids into account. The hierarchical clustering result serves as a reference for relabeling individual clusterings (Nguyen and Caruana 2007). Relabel accuracy is calculated for each subject to identify the permutation of cluster ids that most accurately reflects the match of a given clustering with the reference clustering. Relabel accuracy is also presented as one of the various validity metrics (for more information see Online Resource Sect. 2.4 Relabeling strategy). Next, the *mode* of the relabeled subject-wise clustering is computed and used as the group-level clustering. Optionally, the hierarchical clustering result can be used as a group-level clustering in lieu of the *mode* group-level clustering. Subject-wise follow-up analyses done in subsequent steps refer to the individual clusterings, and group analyses refer to the group-level clustering. Note that the group-level parcellation is not calculated when parcellation is performed in the native space.

### Clustering validity

Finding the appropriate number of clusters is a challenging and unresolved problem. It is common to probe a range of *k* values starting at two to a number determined through prior knowledge of the ROI and the source data (i.e., based on modality, granularity of the data, or the selected target regions) structure. Therefore, the clustering solutions at different *k* need to be evaluated. One possibility is using external validation, which contrasts the clustering solutions against a predetermined structure which is independent of the source data (i.e., using a predefined cytoarchitectonic parcellation as an external reference for clustering the results of the same region). In the absence of information for external validation (as is frequently the case), internal cluster validation can help to select an optimal clustering in a data-driven way. Several such metrics can be used to rank the clustering results. However, different metrics often produce divergent results. Baarsch and Celebi (2012) evaluated the Dunn index, the Davies–Bouldin index, the Calinski–Harabasz index, the Silhouette index, the point biserial measure, the Pakhira–Bandyopadhyay–Maulik (PBM) score, and sum-of-squares. They concluded sum-of-squares to be most effective, closely followed by the Silhouette index. Popular alternatives like the Davies–Bouldin index and the Calinski–Harabasz index were only moderate contenders, while the Dunn index performed poorly. Even the best measure, however, was only correct in 60% of the test cases. Nevertheless, validity metrics are often tested on simulated or simple data sets which might not generalize to the complexity inherent in the connectivity data. Furthermore, there exist many more validity metrics [such as the *I* index, which was tested to perform well in a review by Maulik and Bandyopadhyay (2002)]. In general, it is difficult to deem any single validity metric to be good for clustering, as data properties may vary significantly between data sets. Therefore, *CBPtools* provides several validity metrics and sufficient care must be given when deciding which measure to rely upon, also evident from our results.

#### Validity metrics and report

Clustering outputs the solution for each clustering granularity *k* both separately for each subject and grouped together into a group-level clustering. For each individual clustering at each value of clustering granularity *k*, several cluster quality metrics can be obtained. These include: the Silhouette index (Rousseeuw 1987), the Calinski–Harabasz index (Calinski and Harabasz 1974), and the Davies–Bouldin index (Davies and Bouldin 1979). For group labels, again for each *k*, relabel accuracy and cophenetic correlation, as a measure on how well the pairwise distances between the individual cluster labels are preserved in the group-level clustering, are given. Similarity between individual clusterings can also be examined based on the Adjusted Rand index (Rand 1971; Hubert and Arabie 1985), the V measure (Cramér 1946), or the adjusted mutual information (Vinh et al. 2010) scores (whichever is chosen by the user). The resulting similarity matrices, showing the similarity between pairs of individual subject clusterings for each *k*, are presented as dendrograms. Likewise, similarity between individual clusterings to the group clustering is computed using the same metric. Care needs to be taken when interpreting this score, however, since the group-level clustering is not independent of the individual subject clusterings which might inflate similarity scores. Lastly, the group clustering is mapped upon the ROI mask and stored as an NIfTI image which can be visualized using any of the various NIfTI format image viewers [e.g., Mango (http://ric.uthscsa.edu/mango/), MRIcron (https://www.nitrc.org/projects/mricron) or FSLeyes (https://fsl.fmrib.ox.ac.uk/fsl/fslwiki/FSLeyes)].

The statistics are stored as tab-separated files as well as figures in a user-defined file format. Interim data (e.g., connectivity, cluster labels) are stored as NumPy (Oliphant 2006) binary files and tab-separated text files. This data can then be used to determine what cluster solution best fits the input data, and hence is best suited for further use in more in-depth analyses. Optionally, subject-specific reports (i.e., metrics and plots) can be obtained in addition to the group-level clustering reports if specified in the configuration file (see Online Resource Sect. 1.3.3 Single-subject parcellation). Lastly, one or more reference NIfTI images can be provided to allow direct comparison between the *CBPtools* group-level cluster solutions and a priori parcellations (see Online Resource Sect. 1.3.5 Using reference images).

### Example data

**Fig. 2:**
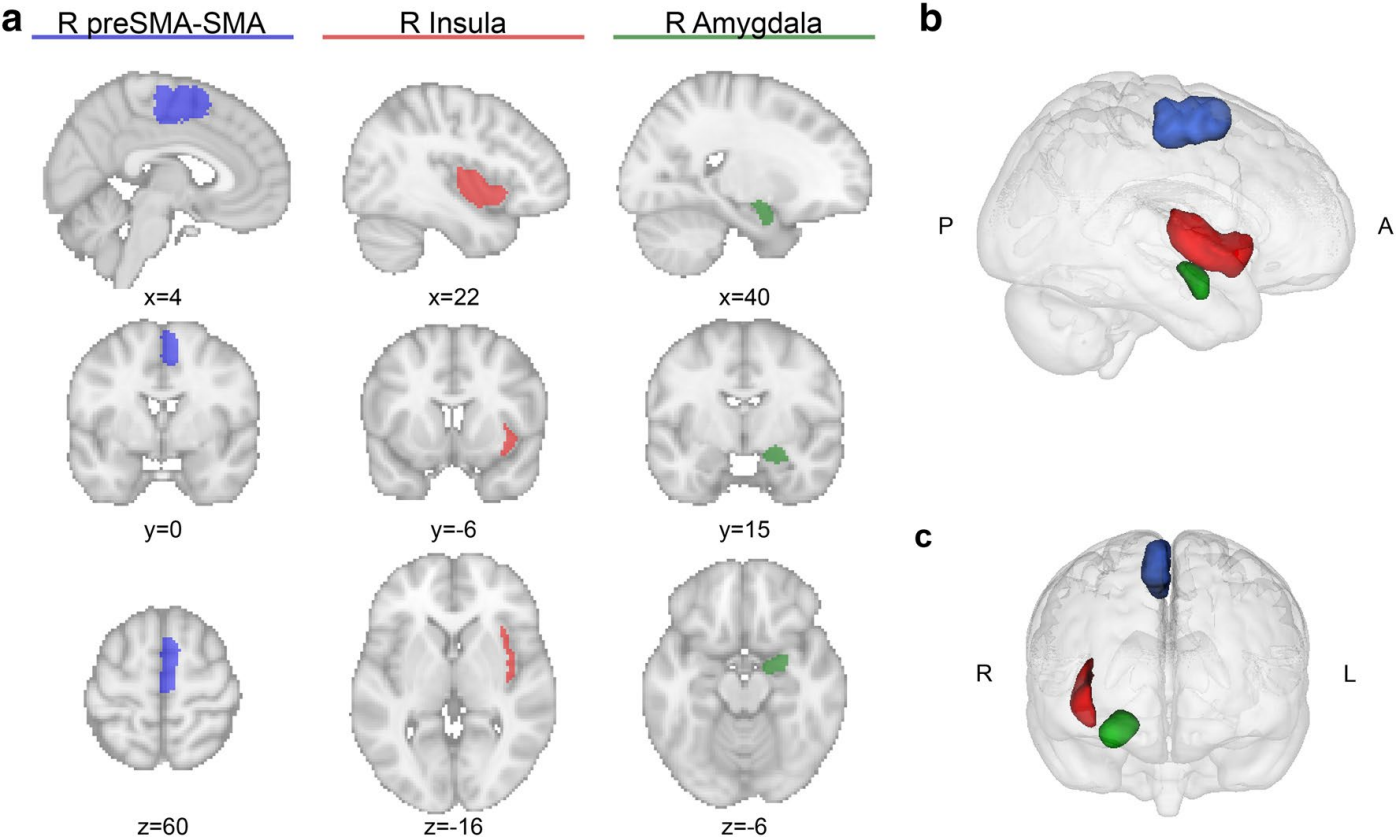
Outline of the three ROIs used for the example procedure. **a** The three columns highlight the R preSMA–SMA (blue, left), R amygdala (green, middle), and R insula (red, right) in sagittal, coronal, and axial (top to bottom) sections. The figures were generated using Nilearn’s plotting tools (Abraham et al. 2014). **b** All ROIs shown from a right-sided view with posterior (P) to the left, and anterior (A) to the right. **c** An anterior view of the three ROIs, with right (R) and left (L) flipped to radiological display convention. The 3D representations in **b** and **c** were generated using Mango (multi-image analysis GUI; http://ric.uthscsa.edu/mango/)

The right (R) preSMA and SMA, R insula, and R amygdala are prominently featured regions in CBP analyses, and were therefore selected as ROIs to evaluate our software (see Fig. 2). The R preSMA–SMA region (at 972 voxels) was extracted using the Juelich Cytoarchitectonic Atlas (Eickhoff et al. 2005; Ruan et al. 2018), and the R insula (546 voxels) and R amygdala (280 voxels) regions were both extracted using the FSL distributed Harvard-Oxford Atlas. The FIX-denoised rsfMRI data of 300 healthy unrelated subjects (mean age 28.57, 150 females, no significant age ($t=0.71$, $p=.48$) and educational ($t=-\,0.31$, $p=.75$) difference between genders) from the HCP (Van Essen et al. 2013), and BEDPOSTX results of the minimally processed (Glasser et al. 2013) dMRI data of the same 300 subjects were used as input data for the rsfMRI and dMRI modalities, respectively.

Workflow execution proceeded separately for each ROI and modality, as depicted in Fig. 1. For each execution the average MNI152 T1 brain (2 mm isotropic) from FSL (Jenkinson et al. 2012) was binarized and used as a whole-brain gray matter target mask. For rsfMRI only, the target mask was subsampled (see "ROI mask preprocessing") to improve computational efficiency.

Including the preprocessing of the ROI and target masks, all the following steps were done by *CBPtools* with the configuration parameters outlined below (these are the *CBPtools* default configuration parameters). The rsfMRI BOLD time series were 5 mm FWHM smoothed, global WM, global CSF, and 24 motion parameter signal corrected (including a bias term), and 0.01–0.08 Hz band-pass-filtered (see the green boxes in Fig. 1c). Global WM and global CSF nuisance signal regression in addition to FIX-denoising was used as it appears to give the highest reliability for rsfMRI CBP (Plachti et al. 2019). The linear correlations between ROI and target voxel time series were then computed to obtain a ROI-to-target connectivity matrix for each subject and Fisher’s *Z* transformed. To derive dMRI connectivity, probabilistic tractography was performed with the following parameters: distance threshold = 5, loop check = true, curvature threshold = 0.2, step length = 0.5, number of samples = 5000, steps per sample = 2000, correct path distribution for pathway length = true. This yielded a high-resolution ROI to low-resolution target (whole-brain) connectivity matrix per subject which was cubic transformed.

Each subject’s connectivity matrix was used as input for *k*-means clustering (with *k* from 2 to 5, the *k*-means++ initialization method, 256 initializations [as suggested by Nanetti et al. (2009), and a maximum of 10,000 iterations; Fig. 1d]. The range of *k* was chosen after consulting relevant literature regarding the three ROIs. To maintain the same settings for each ROI and make replication of the example procedure computationally less intensive, we chose to keep the range of *k* consistent between ROIs. To obtain a group-level clustering, hierarchical clustering with complete linkage and Hamming distance was applied (Fig. 1f) on individual-level clusterings to obtain a combined reference clustering per *k* (Nguyen and Caruana 2007). This reference clustering was subsequently used to relabel the individual clusterings. The resulting labels were used to calculate the *mode* for each voxel, serving as the group-level clustering result for each value of *k*. Cluster validation was performed on the individual clusterings using the Silhouette index, the Calinski–Harabasz index, and the Davies–Bouldin index (Fig. 1e). The adjusted rand Index (ARI) was computed as a similarity measure between individual and group clusterings (Fig. 1g).

The results section is structured such that each ROI reflects a different aspect of the *CBPtools* workflow. For the preSMA–SMA ROI we highlighted the reproducibility of histological parcellations, for the insula we focussed on the subdivisions of various *k* cluster solutions for the group parcellations and, lastly, for the amygdala we evaluated the cluster validity metrics provided as output by the workflow. All output not highlighted here is available in the Online Resource.

## Results

### preSMA–SMA parcellation

**Fig. 3:**
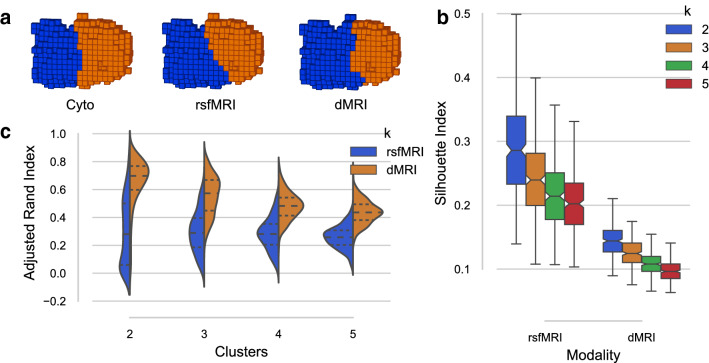
R preSMA–SMA results from the rCBP procedure. **a** The two-cluster solutions of the combined R preSMA and SMA ROI for the cytoarchitectonically defined (Ruan et al. 2018) subdivision from the Jülich histological atlas (Eickhoff et al. 2005), and the rsfMRI and dMRI connectivity-based parcels from left to right. The 3D representations were generated using matplotlib’s 3D voxel/volumetric plotting and are in the same view as Fig. 2b. **b** ARI scores between the individual subject clustering results and the group-level clustering result for both rsfMRI and dMRI for $k = [2, 3, 4, 5]$. **c** Silhouette index for all cluster solutions ($k = [2, 3, 4, 5]$) where a higher Silhouette index indicates a better fit. Here, the two-cluster solution seems to best fit the input data for both rsfMRI and dMRI

The group clusterings for the two-cluster solution approximated the R preSMA–SMA cytoarchitectonic differentiation with an ARI of .71 for rsfMRI, and .76 for dMRI results (where 0 indicates no similarity at all, and 1 indicates perfect similarity). That is, only 76 (7.82%) and 63 (6.48%) out of all voxels were mismatched for rsfMRI and dMRI, respectively. Figure 3a provides a visual representation of the ROI with the two-cluster labels mapped onto it for the cytoarchitectonically defined region, and the two rCBP defined subdivisions using rsfMRI and dMRI data. The Silhouette index (Fig. 3c), Davies–Bouldin index, and Calinski–Harabasz index all indicated the two-cluster solution as the best fit to the rsfMRI input data (note that the Davies–Bouldin index indicates a better fit through a lower value). The Silhouette and Calinski–Harabasz indices obtained from the dMRI clusterings both suggested the two-cluster solution, with only the Davies–Bouldin index suggesting a slightly better fit for the three-cluster solution. Our results are consistent with previous studies regarding functional and structural parcellation of the preSMA–SMA regions (Johansen-Berg et al. 2004; Klein et al. 2007; Kim et al. 2010; Zhang et al. 2015).

### Insula parcellation

**Fig. 4:**
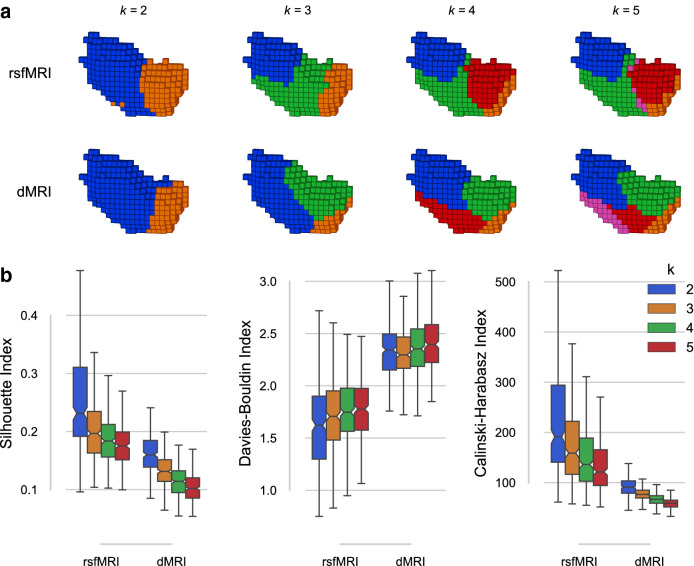
R Insula results from the rCBP procedure. **a** Insula parcels for the two-, three-, four-, and five-cluster solutions obtained from rsfMRI (top row) and dMRI (bottom row) connectivity. All images are in the same view (right-sided) as Fig. 2b. **b** Internal validity scores for all tested solutions ($k = [2, 3, 4, 5]$). The Silhouette index (left) and the Calinski–Harabasz index (right) indicate a better fit through a higher score, whereas the Davies–Bouldin index (middle) is better when lower

All internal validity metrics agreed that a two-cluster separation into an anterior and posterior subdivision fitted the rsfMRI source data best. The two- to five-cluster solutions are shown as 3D volumetric/voxel plots in Fig. 4. The three-cluster rsfMRI solution added a medial parcel (green), extending more into the anterior parcel (blue) rather than the posterior parcel (orange) of the two-cluster solution. The four-cluster rsfMRI solution further subdivided the medial and part of the posterior parcel into dorsal-anterior and medial parcels (green and red, respectively), whereas the five-cluster solution added only a thin parcel (magenta) in between the aforementioned dorsal-anterior and medial parcels.

Likewise for dMRI data, the two-cluster solution separated the R insula into anterior and posterior subdivisions. However, the Davies–Bouldin index slightly diverged from the other metrics, instead suggesting a three-cluster solution to best fit the source data. The shape of the dMRI clusters also showed a different picture than the rsfMRI results, particularly for the three- and five-cluster solutions. The three-cluster solution added a medial parcel that did not extend as much into the dorsal direction as the rsfMRI three-cluster solution did. Slightly more agreement between modalities was found in the four-cluster solution, where the posterior parcel was subdivided into a dorsal (blue) and ventral (red) part, the latter of which was further split into the five-cluster solution.

Functional parcellation of the two-cluster rsfMRI solution for the insula was in line with prior parcellations (Kelly et al. 2012). In addition, Nanetti et al. (2009) suggest a common parcellation of the insula along the anterior-posterior axis for dMRI data. The four-cluster dMRI parcellation furthermore visually resembles the insula’s functional differentiation uncovered by Kurth et al. (2010b) using a meta-analytic approach, with only our ventral-anterior parcel (red) extending more anteriorly than theirs.

### Amygdala parcellation

**Fig. 5:**
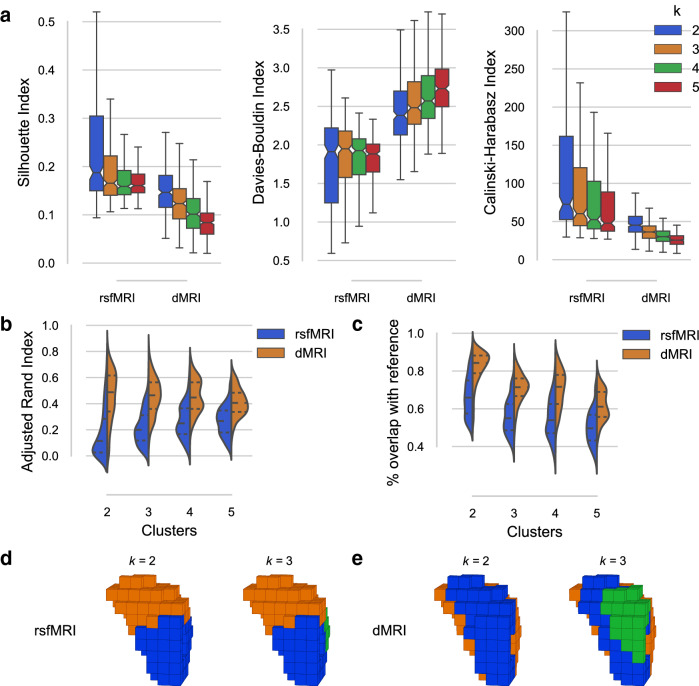
Various *CBPtools* output figures for the R amygdala parcellation. **a** Internal validity scores for all tested solutions ($k = [2, 3, 4, 5]$). The Silhouette index (left) and the Calinski–Harabasz index (right) indicate a better fit through a higher score, whereas the Davies–Bouldin index (middle) is better when lower. **b** Group similarity scores (i.e., the similarity of individual clusterings to the group clustering), with the cluster number *k* on the *x*-axis, comparing rsfMRI (blue) to dMRI (orange). **c** Relabel accuracy displayed in a similar format as b. **d** The two- and three-cluster solution of the R amygdala for rsfMRI. **e** the same for dMRI

Similar to the other two ROIs, the model that best fits the data in the R amygdala was bipartite. Nevertheless, the two-cluster solutions for rsfMRI and dMRI connectivity differed substantially (Fig. 5d, e). On the one hand, the rsfMRI two-cluster solution showed a dorsal (superior) and ventral (inferior) subdivision of the Amygdala. On the other hand, the dMRI two-cluster solution showed a medial and lateral subdivision. At higher clustering granularities clusters split further among the aforementioned axes, rather than finding common ground.

The Silhouette and Calinski–Harabasz indices (Fig. 5a) suggested a two-cluster solution to best fit the rsfMRI source data. However, the Davies–Bouldin index instead suggested a five-cluster solution to fit better. The two-cluster solution approximated prior functional parcellations of the amygdala (Mishra et al. 2014; Zhang et al. 2018) and also prior cytoarchitectonic mapping of the region (Amunts et al. 2005). The dorsal cluster (orange) overlapped with the cytoarchitectonic outline of R amygdala centromedial and amygdalostriatal subregions, whereas the ventral cluster (blue) overlapped with the laterobasal and superficial subregions. At the three-cluster granularity, the ventral cluster was divided into a cluster resembling the cytoarchitectonic laterobasal subregion (blue) and one resembling the superficial subregion (green), the latter of which is best seen from a left-sided view (Fig. S6b). The four-cluster solution subdivided mostly the dorsal cluster (orange), with the new cluster resembling the amygdalostriatal subregion. However, it appeared far larger than its cytoarchitectonic counterpart. The five-cluster solution further subdivided the ventral cluster (blue), but here no further cytoarchitectonic subdivisions exist.

For the dMRI clusterings, all validity indices (Fig. 5a) suggested a two-cluster solution to best fit the source data. As the clustering granularity increased, the R amygdala split further along its medial-lateral axis. Previous parcellation works using dMRI data (Solano-Castiella et al. 2010; Saygin et al. 2011; Fan et al. 2016; Wen et al. 2016) also found similar clusters along the medial-lateral axis. ARI similarity of individual clusterings to the group-level clustering (Fig. 5b) was higher for clusterings on dMRI data than on rsfMRI data, also reflected by the relabel accuracy (Fig. 5c).

## Discussion

### General pattern revealed by CBP

Separating the SMA and preSMA is a popular approach to validate rCBP methods (Johansen-Berg et al. 2004; Klein et al. 2007; Kim et al. 2010; Zhang et al. 2015), as it provides a gold standard and furthermore highlights the ability of the rCBP procedure to reproduce histological parcellations. These two neighboring regions exhibit an abrupt change in connectivity profile where their borders are expected to be, attributed to predominant connections to the motor regions for the SMA and prefrontal connections for the preSMA (Johansen-Berg et al. 2004). As voxels are assigned to clusters based on similarity in their connectivity profiles, separating the preSMA–SMA ROI through automated parcellation approaches should therefore be straightforward. By using the cytoarchitectonically defined preSMA and SMA regions as external validation, we were able to assess histological reproducibility of the preSMA–SMA ROI using *CBPtools*. This was achieved with a very high similarity (ARI $>0.7$) for both the dMRI and the rsfMRI connectivity-driven parcellation to the cytoarchitectonic definition of the ROI.

While results for the preSMA–SMA parcellation were rather straightforward, this was not the case for the R insula parcellation for which many different suggestions for optimal cluster solutions exist in the literature (two-cluster (Cauda et al. 2011), three-cluster (Deen et al. 2011; Chang et al. 2013), and four-cluster (Kurth et al. 2010b), as well as various solutions exceeding our *k*-range (Kelly et al. 2012)). These differences may in part be caused by relatively small data sets with different properties, difficulties on account of intersubject alignment when delineating the ROI mask, as well as variability between research groups in their implementation and use of methods and imaging modalities. Our results suggested a two-cluster solution to best fit both the dMRI and rsfMRI-based connectivity data, although this does not imply it is neurobiologically optimal. Early work on the insula has provided evidence for an anterior (dysgranular) and posterior (granular) subdivision separated by the central insular sulcus (Brodmann 1909). Cytoarchitectonically the posterior insula can be further subdivided into two dorsal posterior areas and one ventral posterior area (Kurth et al. 2010a), but no evidence exists for the anterior insula. Whereas the two-cluster solution matched well between the dMRI and rsfMRI modalities, the results diverged at the three- and five-cluster granularities. The mid-posterior cluster appearing in the dMRI four-cluster solution (Fig. 4a; red) and the mid-anterior cluster appearing in the rsfMRI four-cluster solution (red) made the solutions at the four-cluster granularity more similar. However, the rsfMRI mid-posterior cluster (green) extends more dorsally than its dMRI counterpart. Meta-analysis of the insula (Kurth et al. 2010b) resembles the four-cluster solution of the R insula, associating the posterior cluster (blue) with sensorimotor function, the ventral-anterior cluster (orange) with social-emotional functions, the dorsal-anterior cluster (green) with cognitive functions, and the medial cluster (red) with chemical sensory functions. The medial cluster extends further into the posterior direction for the dMRI parcellations than is the case for the meta-analytic results, and in addition extends further dorsally for the rsfMRI parcellation.

For the R amygdala, the two-cluster solution best represented the data for both the dMRI and rsfMRI modalities. The rsfMRI parcellation of the R amygdala that best fit the source data was a bipartite dorso-ventral subdivision. These results match earlier findings of Mishra et al. (2014), likewise a dorso-ventral (superior–inferior) subdivision in the two-cluster solution using functional connectivity, and a similar dorsal, ventral, and medial subdivision for the three-cluster solution. The same three-cluster solution was found by Zhang et al. (2018). Our validity metrics indicated a best fitting two-cluster solution, but as this does not necessarily imply neurobiological accuracy, a three-cluster solution is likewise viable. Furthermore, the parcellations visually correspond to the cytoarchitectonic mapping of the R amygdala (Amunts et al. 2005) up to the four-cluster solution.

Where a bipartite dorso-ventral subdivision of the amygdala best fits the rsfMRI data, the dMRI data instead best fits within a bipartite medio-lateral subdivision. This pattern resembles the two-cluster solution found by Solano-Castiella et al. (2010) and Fan et al. (2016) in that the solution divided the amygdala into a medial and a lateral cluster. Tract tracing of the rat amygdaloid complex shows that the medial amygdala is related to connections between both intrahemispheric amygdalae (Pikkarainen and Pitkänen 2001). The lateral amygdala is instead found to be connected to somatosensory cortical areas (Jolkkonen and Pitkänen 1998). Solano-Castiella et al. (2010) note the possible existence of a third cluster between the medial and lateral clusters, which resembles the pattern of clusters we found for the three-cluster solution.

The amygdala is a peculiar region on account of its spatial location, which may explain the differences between the rsfMRI and dMRI results. Whereas the rsfMRI parcellations resemble cytoarchictural subdivisions, the dMRI results may instead be driven by spatial artefacts on account of false positives in probabilistic fiber bundle tracking (Zalesky et al. 2016; Maier-Hein et al. 2017) of subcortical areas. As the region gets split at higher granularities, it is possible that instead of creating subdivisions on the basis of neurobiologically relevant signals, instead the subdivisions are driven by noise in the signal on account of methodological idiosyncracies. Investigating why such subdivisions occur at higher granularities is beyond the scope of this work, but is nonetheless an important consideration when investigating clusters with dMRI data. We further investigated whether parcellations of the R amygdala were driven by within-ROI and short-range connections. We parcellated dMRI data after excluding ROI-to-ROI connectivity (excluding 5 mm, 20 mm, and 40 mm border around the ROI in Online Resource Fig. S7), including a mapping of the linear correlations predominantly driving the parcellation results (Online Resource Fig. S9). The resulting parcellations remained mostly unchanged, exhibiting the same medio-lateral pattern of subdivisions.

Overall, divergence between validity indices (i.e., the Davies–Bouldin index from the other validity metrics) highlights the importance of choosing a proper validity metric, each of which assesses cluster validity in a unique way. Note that comparisons of validity scores outside of the sample are meaningless, hence rsfMRI and dMRI validity scores cannot be directly compared. For the R insula the divergence is not necessarily surprising, as it shows transitional changes in cytoarchitecture (Kurth et al. 2010a), rather than sharp cytoarchitectonic borders present between the preSMA and SMA, making it difficult to define stable hard borders. Similarity of cluster labels between subject-wise clusterings may vary considerably. It is as of yet unclear what factors contribute to the high dissimilarity between subjects on some cluster solutions and for some ROIs. For instance, similarity values for the individual clustering results to the group-level clustering results on the two-cluster preSMA–SMA solution are high (see Online Resource Fig. S4b), which may imply that the regions have strongly divergent connectivity patterns that are stable between subjects. However, regions such as the amygdala show lower similarity values. This may in part be due to poor signal to noise ratio with MRI in the subcortical regions (Noble et al. 2017). Nevertheless, the solutions showcased here can be found in previously published works. However, as far as we are aware, no data-driven clustering was performed using dMRI data for the R amygdala at higher granularities.

### Conclusions and perspectives

Here, we have demonstrated the effectiveness of using *CBPtools* to procure resting-state functional and diffusion MRI connectivity-derived parcellations on three functionally and spatially different ROIs. Connectivity and clustering methods have been carefully chosen to both reflect the most popular and the most widely evaluated approaches in the brain mapping community. The procedure is customizable through a configuration file, allowing for fine-tuned processing for each ROI. Furthermore, by providing or specifying input as well as parameters given to *CBPtools*, any parcellation work can be reproduced with relative ease and, importantly, can be compared to other works using this tool. To illustrate the efficiency of the procedure, we have provided benchmarks (see Online Resource Sect. 3.1 Benchmarks) as a guideline for what to expect when executing *CBPtools* on a similar data set, with similar settings, on an average computational cluster. Through the use of the *CBPtools* output, a user will be able to quickly generate parcellations and validity metrics that can either be used directly or used to inform a more detailed post hoc analysis. For instance, the selection of clustering granularity as well as multi-modal integration of cluster solutions may require further fine-grained and region-specific analyses. We opted to provide all *k* cluster solutions with guidance for the user to choose the optimal solution, as there likely is no ’one true parcellation’, but instead biologically relevant maps at different granularities.

Future development of the software will support the integration of MACM and structural covariance modalities, found to be valuable for studying the brain and adding an additional layer of information to multi-modal CBP (Eickhoff et al. 2015; Plachti et al. 2019). In the framework of the Human Brain Project and in collaboration with the Juelich Supercomputing Center, a web-based version of the software that can execute the rCBP procedure on various predefined and preprocessed large data sets is planned. This will offer a high-throughput solution for massive parallelization for rCBP on a user-defined ROI, in an online environment where all summary output will be available for download.

In summary, we provide an openly distributed package for performing rCBP for which to our knowledge there is currently no alternative. We have outlined its procedure and demonstrated its efficacy using three commonly parcellated ROIs on a substantial data set. By introducing *CBPtools* we provide researchers the means to conduct reproducible, data-driven rCBP analyses on multiple neuroimaging modalities and large amounts of subject data.

## Electronic supplementary material

Below is the link to the electronic supplementary material.

Supplementary material 1 (PDF 2874 kb)
