# Supplementary material for: CBPtools: a Python package for regional connectivity-based parcellation
Source: Brain Struct Funct. 2020 Mar 6;225(4):1261–75. doi: 10.1007/s00429-020-02046-1 (PMC7271019; doi:10.1007/s00429-020-02046-1)
Supplement: Supplementary file 1 — Supplementary material 1 (PDF 2874 kb) [file 429_2020_2046_MOESM1_ESM.pdf]

CBPtools: A Python package for regional connectivity-based parcellation

Niels Reuter[1,2], Sarah Genon[2], Shahrzad Kharabian[2], Felix Hoffstaedter[1,2], Xiaojin Liu[1,2], Tobias Kalenscher[3], Simon B. Eickhoff[1,2], and Kaustubh R. Patil *[1,2]

[1] *Institute of Systems Neuroscience, Heinrich-Heine University, Düsseldorf, Germany*
[2] *Institute of Neuroscience and Medicine (INM-7), Research Centre Jülich, Jülich, Germany*
[3] *Comparative Psychology, Institute of Experimental Psychology, Heinrich-Heine University Düsseldorf, Düsseldorf, Germany*

January 14, 2020

1 CBPtools instructions

For a detailed manual on using *CBPtools*, please visit the online documentation (Reuter, 2019).

1.1 Installation

Python version 3.5 or higher is required to install *CBPtools*. All dependencies will be installed automatically along with *CBPtools*, except for FSL's *PROBTRACKX2* which must be installed manually (see https://fsl.fmrib.ox.ac.uk/fsl/fslwiki/FslInstallation). This tool is required to perform probabilistic tractography on diffusion-weighted imaging data, making it only necessary when diffusion magnetic resonance imaging (dMRI) data is used. It is recommended to use a dedicated virtual environment (see https://packaging.python.org/guides/installing-using-pip-and-virtual-environments/). To install *CBPtools*, use the following pip command:

```
pip install cbptools
```

1.2 Usage example

For the following example we have supplied preprocessed resting-state functional magnetic resonance imaging (rsfMRI) and dMRI data for 100 randomly drawn subjects out of the 300 subjects described in the Example data section of the paper (mean age 28.46, 50 females, no significant age (t=-1.5, p=0.14) and education (t=-1.04, p=.30) difference between genders). We have also included the three region-of-interest (ROI) NIfTI images used in the paper, as well as the *CBPtools* configuration files used to process the data. The example data set was prepared and uploaded using DataLad version 0.12.0rc6 (Halchenko et al., 2019b) and has a total size of 243GB. The example data can be downloaded using DataLad (Halchenko et al., 2019a), which can be installed using *apt-get* or *pip* (note when using *pip*, the *git-annex* dependency must be installed manually; see https://www.datalad.org/get_datalad.html). The example data is located on a remote location linked to through GitHub (https://github.com/inm7/cbptools-example-data).

```
datalad install --get-data --source https://
    github.com/inm7/cbptools-example-data.git
```

Further downloading options (i.e., downloading only parts of the data) are outlined in the online documentation (Reuter, 2019).

A *CBPtools* project can be created using any of the provided configuration files or a custom configuration file. In this example, we will use the preSMA-SMA ROI with the rsfMRI data (i.e., the '*config_r_presma-sma_rsfmri.yaml*' configuration file), although it can be substituted by any other configuration file to use different settings and data.

```
cd cbptools-example-data
cbptools create --config config_r_presma-
    sma_rsfmri.yaml --workdir /path/to/workdir
```

The *workdir* parameter is used to define where the project files (and eventual output data) will be stored. This can be any directory on the file system with read and write access.

Any errors and warnings occurring during the setup will be logged. The log is available either in the current directory (if the setup fails) or in the log folder inside the *workdir* (if the setup succeeds). If there are any errors, the project will not be created until they are resolved. If there are no validation problems, the project will be created. Change directory to the *workdir* and execute the workflow (contained in the *Snakefile*) using Snakemake, which is installed as a dependency of *CBPtools*.

*Corresponding author: k.patil@fz-juelich.de

```
cd /path/to/workdir
snakemake
```

For more customization of the Snakemake execution, visit the Snakemake guide (Köster, 2019) or the execution section of the *CBPtools* online documentation.

1.3 Alternative settings

There are various ways to configure *CBPtools* either to process data differently, or to receive different outputs. In the paper, the focus was primarily on processing the dMRI and rsfMRI modalities to obtain the group-level parcellation results. Alternatively, users may be interested in using multi-session data or single-subject parcellations.

1.3.1 Clustering algorithms

The k-means algorithm is the default clustering algorithm in *CBPtools* and was used to obtain the results in the paper. However, *CBPtools* also provides agglomerative (hierarchical) and spectral clustering as options to obtain subject-level parcellations. The method to obtain group-level parcellations remains the same irrespective of subject-level parcellation algorithm.

All three available algorithms (k-means, agglomerative clustering and spectral) use the scikit-learn package (Pedregosa et al., 2011). To be more precise, the implementations are the *KMeans*, *AgglomerativeClustering*, and *SpectralClustering* classes from the *sklearn.cluster* module. The parameters that can be specified for each of these algorithms can be adjusted in the configuration file. Note that not all scikit-learn provided parameters can be used, as some are not relevant for CBP or do not work in conjunction with the other steps in the workflow. Importantly, the clustering results strongly depend on the parameters used. While *CBPtools* has a set of default parameters, they might not be the best choice for a given ROI. For example, it is possible that the spectral clustering algorithm might not be able to properly compute a (semi-)positive definite similarity matrix when inappropriate kernel parameters are used. In this case the spectral clustering cannot proceed. For such cases, *CBPtools* will log the issues that occur during the clustering step, perform clustering for all possible subjects, and then halt processing until the issues are resolved.

We would like to note that even if clustering succeeds, the clustering results may not necessarily reflect biologically plausible results. For instance, a 2-cluster solution may assign only one voxel to a cluster, and all other voxels to the other cluster. If this happens, the group-clustering (when *mode* is being used as the method) may entirely remove the small cluster, resulting in a 1-cluster solution being posed as a k-cluster solution. We therefore strongly suggest investigating the single-subject cluster labels and not only the group-level clustering results. It is highly recommended to examine the log files to identify any problematic runs and parameters causing them.

When using the connectivity input modality with spectral clustering, it is possible to provide adjacency instead of connectivity matrices. When choosing this option, the adjacency matrices are given as input (in the configuration field **data: connectivity**) and the spectral clustering affinity must be set to precomputed (**parameters: clustering: cluster_options: kernel**). This is not possible with k-means or hierarchical clustering.

1.3.2 Multi-session input data

Multi-session data (i.e., a data set with multiple runs per subject) can be processed using *CBPtools* by specifying sessions in the configuration file. Data for each session will be processed separately until the connectivity step (Fig. 1c), after which the connectivity matrices for each subject will be averaged across all the sessions. When using multi-session data, the (optional) PCA transformation will be performed after averaging the connectivity matrices, whereas the other transformations (Fisher's Z transform and cubic transform) will be applied before averaging. The averaged connectivity matrices will then be used for the remainder of the procedure.

1.3.3 Single-subject parcellation

CBPtools can generate subject-specific reports (i.e., metrics and plots) in addition to the group-level clustering reports if specified in the configuration file. This is done by setting the field **parameters: report: individual_plots** to true. This option is turned off by default as it requires more computation time.

CBPtools can also be used to obtain parcellations in the subject's native space by setting the **data: masks: space** field to 'native'. In this case all the data, i.e., the input masks and the fMRI and dMRI data, should be in the native space. Furthermore, the target mask is no longer optional, as the default target mask is in a common reference space rather than native space. Note that group-level parcellations cannot be computed in this scenario. This is because *CBPtools* does not perform any transformations to bring native data into a common reference space. As a result, only the subject-level parcellations can be computed. When subject-specific input masks are provided, *CBPtools* will generate all figures for each individual subject, rather than generate output at the group level. Steps F and G in Fig. 1 are skipped, and step H will provide different output. Note that in any case, seed and target masks must always be in the same space.

1.3.4 Automatically deriving the seed mask from an atlas

To accommodate the use of atlases, an atlas can be provided as a seed mask (i.e., in the **data: masks: seed**

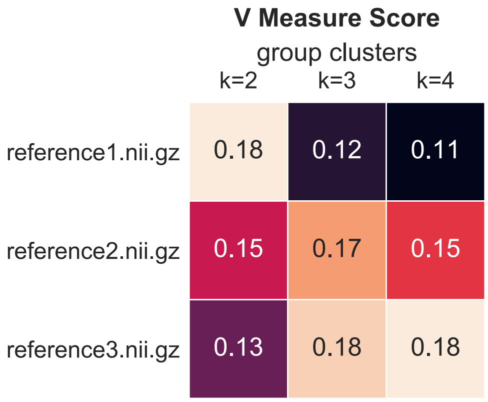

Fig. S1 Reference similarity heatmap example. Three different group clustering results ($k = [2, 3, 4]$) are compared to three different reference NIfTI images. Note that the reference images as well as the group cluster results are derived from dummy data, purely for illustration purposes.

field) instead of a binary seed mask. When doing so, the atlas must be a NIfTI image using integers as region-ids, and the region(s) to be used as a seed mask must be specified as one or more region-ids (integers) in the configuration file. A composite binary mask of the specified region(s) will be generated. Since *CBPtools* doesn't perform any image warping, the atlas has to be in the same space as the input data (i.e., the time-series for the rsfMRI modality, and the bedpostX output for the dMRI modality).

1.3.5 Using reference images

To allow direct comparisons between the *CBPtools* group-level cluster solutions and *a priori* parcellations (e.g. the cytoarchitectonically defined preSMA-SMA subdivision as used in the paper) one or more reference images can be provided. These images must be in the same space as the seed mask, covering the same voxels, and have at least two clusters. A similarity score (either Cramer's V measure, adjusted Rand index, or adjusted mutual information score) will be computed between each reference image and each group clustering solution. The output will be provided as a tab-separated file, as well as a heatmap. An example of the latter is shown in Fig. S1.

1.4 Running multiple CBPtools projects in parallel

Clustering of input data from the rsfMRI and dMRI modalities are run separately (i.e., no multi-modal solution is provided), and the various configuration options can be different between the modalities. *CBPtools* cannot create a project that processes both rsfMRI and dMRI data within a single workflow. However, it is possible to create multiple projects (using different configuration files) to subsequently run them in parallel by executing snakemake once per project. On a system that is not managed by a scheduler (e.g. SLURM, qsub, HTCondor, etc.) it is recommended to specify the number of jobs and amount of memory each snakemake instance can use. If a scheduler is being used, this is not necessary. For example, on a system with 8 threads and 30 GB of memory the following approach can be used to initialize two *CBPtools* projects (with each having access to half of the available resources):

```
cbptools create -c config_r_presma-sma_rsfmri.yaml -w r_presma-sma_rsfmri
cbptools create -c config_r_amygdala_dmri.yaml -w r_amygdala_dmri
```

Two terminal windows can then be opened, to run the following commands (one in each terminal):

```
cd r_presma-sma_rsfmri
snakemake -j 4 --resources mem_mb=15000
```

and

```
cd r_amygdala_dmri
snakemake -j 4 --resources mem_mb=15000
```

2 Supplementary methods

2.1 Connectivity

For the rsfMRI modality, connectivity is calculated using linear correlations between the ROI voxels of the time-series of a subject (x), and the target voxels of the time-series of the same subject (y). The target can be any part of the brain, although the example data in the paper uses a subsampled whole-brain gray matter mask. Both x and y are first standardized. The correlation is performed by transposing y and taking its dot product of x, then dividing this by the number of voxels in x and transposing the result again. In the event there are voxels without sufficient variance (i.e., variance not exceeding a threshold of the smallest representable number in Python's NumPy package, *np.finfo(np.float32).eps*) within either the target or ROI masked time-series, the standardization will have failed (i.e., a division by zero will have occurred on account of the standard deviation being zero). Hence, all correlations resulting from a computation with a *NaN* (not a number) element are set to zero. If a Fisher's Z transform is to be performed on the connectivity matrices, then values at precisely 1 or -1 will result in an infinite value. We set them slightly below 1 or above -1 in order to prevent this.

2.2 Median filtering

Median filtering is an optional procedure which can be applied to 'clean-up' artifacts in a seed mask. Sometimes, a seed mask contains small holes, single-voxel strands that portrude from the mask, or sharp borders. These

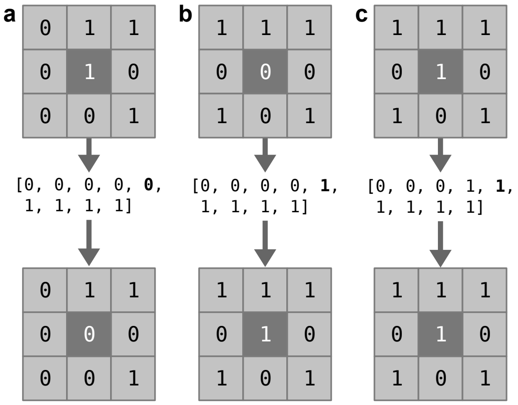

Fig. S2 Median filtering example. The top row shows a selected voxel (center) and its nearest neighbor voxels (off-center), where 1 means the voxel is part of the mask, and 0 means it is not. The view is reduced to 2D for simplicity. The middle row shows all voxel values ordered incrementally. The median value is highlighted in bold. The bottom row shows the same as the top row after the selected voxel has its value changed with the median of itself and its neighbors. **a** Median filtering that results in the voxel being set to 0 (i.e., not part of the mask). **b** Median filtering that results in the voxel being set to 1 (i.e., part of the mask). **c** Median filtering that does not result in any changes (i.e., the voxel remains part of the mask)

artifacts usually arise in hand-drawn ROIs. Median filtering may be a useful tool to get rid of such artifacts. The reason why median filtering is particularly useful for hand-drawn masks is that they usually lack continuity in the 'depth' direction when drawing in 2D. When such artifacts are not expected, e.g. for atlas-derived ROIs, this is not a recommended option. For each selected voxel within the binary mask ROI, median filtering reassesses its selection based on its neighborhood. To apply median filtering, the spatial nearest neighbors are taken for each voxel (resulting in a 3x3x3 matrix of the selected voxel and its neighbors) and the median selection value (i.e., median of all the values in the matrix) is assigned as the new selection value for this voxel. Fig. S2 shows three examples of this procedure simplified to a 2-dimensional space. The first example (Fig. S2a) shows a selected voxel that has too few neighbors that are part of the mask. As a result, the voxel is removed from the mask by having its value set to zero (the median). The second example (Fig. S2b) instead shows a selected voxel that is not part of the mask, but has many neighbors that are part of it. The selected voxel is added to the mask by having its value set to one. Lastly, the third example (Fig. S2c) shows a selected voxel that is part of the mask and has many neighbors that are likewise part of the mask. Its value remains unchanged, as the median is the same as its original value.

2.3 Nuisance signal regression

Nuisance signals (i.e., confounds) can optionally be supplied as a tab-separated file per subject. This file is expected to have a header on the first row naming each column. The column names can be used to select columns to be used as nuisance regressors (note that if no columns are specified, then all columns are used). Commonly used columns may be white matter, cerebrospinal fluid, gray matter, or global signal, as well as motion regressors to correct for the effects of head motion in the scanner. If required, a linear trend and constant should be added as columns to the files. The nuisance signal removal is applied as a linear regression of the confound time-points on the time-series of the corresponding subject and retaining the residuals as the new signal.

2.4 Relabeling strategy

Relabeling is applied to obtain a group-level parcellation by combining subject-level parcellations. The subject-level cluster labels (per k) are obtained by applying the k-means (or, alternatively, agglomerative or spectral) clustering algorithm. This results in a cluster labeling per subject, per k. Fig. S3a shows an example set of labels for $k = 5$. The cluster-ids (represented by numbers and colors) are arbitrary (i.e., they can be permuted), yet in this particular example all subjects have identical clusterings (the dotted line separates the different clusters).

To interpret the parcellations over a population, the parcellations must be combined into a single (group) parcellation per k by computing the most representative cluster assignment for each ROI voxel across subjects. As the cluster-ids per subject are arbitrary, they need to be reassigned such that the most similar clusters between subjects get assigned the same cluster-id. To achieve this, we check all permutations of the cluster-id (Fig. S3d) per subject and compare the permutation-derived labels to a reference clustering representative to all subjects. The permutation most similar to the reference is used to reassign cluster-ids for that subject.

The reference clustering (Fig. S3b) is obtained by performing hierarchical clustering (Fig. S3c) with Hamming distance on all of the subject labels (Nguyen and Caruana, 2007). Hamming distance is insensitive to cluster-id permutations as as it measures the minimum number of substitutions required to change the set of cluster labels from one subject to that of another. Once all subject labels have been relabeled to best match the reference clustering, they become comparable. The most frequent assignment for each voxel is then obtained by taking the *mode* over the relabeled cluster-ids and used as the group level parcellation (Fig. S3e).

Note that in Fig. S3, all subjects clusterings are identical for the assignment of cluster-ids. This is an ideal situation, but not a realistic one. In real-world data, it is common to see differences in relabeled results.

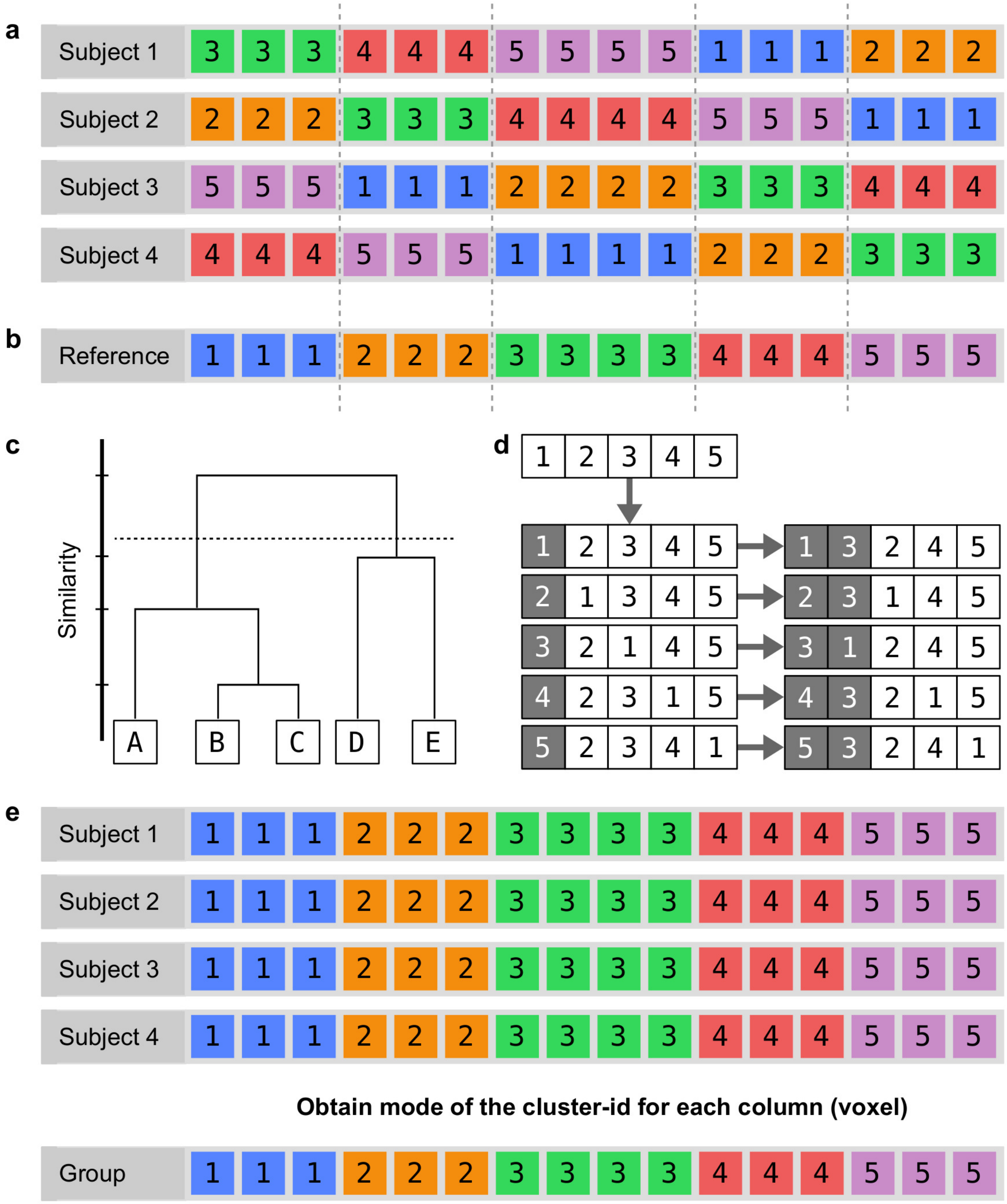

Fig. S3 Relabeling strategy to obtain group cluster labels. **a** Four example label sets obtained from clustering, where each color/number represents a cluster-id that is assigned randomly. Each of the subjects has an identical clustering, yet the cluster-ids differ. **b** Reference cluster labels obtained through hierarchical clustering of the subject labels in a. **c** Illustration of hierarchical clustering performed on 5 voxels. **d** Cluster-ids are swapped for each possible permutation of the cluster-ids array. Each permutation is then tested for similarity against the reference clustering from b. **e** The cluster labels for each subject after the relabeling strategy has been applied. The mode is obtained for each voxel, resulting in the group clustering

3 Supplementary results

For all example ROIs outlined in the paper we have obtained additional results, i.e., validity metrics and cluster solutions. Fig. S4 contains all the results provided as output by *CBPtools* for the preSMA-SMA ROI. While the results in Fig. S4a have been shown in full for the R insula and R amygdala ROIs, Fig. S4b contains the relabel accuracy as described in the paper (Sect. 2.1.5 Individual- and group-level clustering), showing that in particular for the dMRI 2-cluster solution the percentage of overlap is high. Generally, the adjusted rand index (ARI) is more credible than the percentage of overlap as it corrects for chance grouping of voxels within a cluster solution. The cophenetic correlation is displayed in Fig. S4c. It is a measure on how well the pairwise distances between the individual cluster labels (i.e., the cluster labels generated from each subject's connectivity matrix) are preserved in the group-level clustering. In this case it is the *mode* of the relabeled individual clustering results for each value of k, as described in the paper (Sect. 2.3 Example data).

The result is particularly high for the chosen 2-cluster solution for both dMRI and rsfMRI. Fig. S4d shows the remaining 3, 4, and 5-cluster solutions, although both internal and external validity suggests a 2-cluster solution. The same remaining results are presented for the R insula (Fig. S5) and the R amygdala (Fig. S6). Note that in Fig. S6b a left-sided view is provided for some of the clusterings, as the right-sided view used for all ROI representations obscures one or more of the clusters.

3.1 Benchmarks

Benchmarks were obtained using Snakemake's (Köster and Rahmann, 2012) benchmarking utilities. Snakemake uses the *psutil* package to obtain the values for various benchmarking metrics. We have benchmarked the rsfMRI and dMRI workflows for the preSMA-SMA ROI parcellation as described in the paper. The benchmarks were performed on a system with 30 total available threads and 100GB total available random-access memory (RAM). The data set was stored on a shared remote storage server, hence competing for file read and write speed (I/O) may have increased the duration of each individual task. The entire procedure for the rsfMRI data took 1 day, 14 hours, 56 minutes, and 1 second. For the dMRI data, it took 3 days, 23 hours, 42 minutes, and 23 seconds.

Tables 1, 2, 3, 4, 5, and 6 show the total task duration, maximum resident set size (RSS), maximum virtual memory size (VMS), maximum unique set size (USS), maximum proportional set size (PSS), and maximum CPU load in seconds for regional CBP of the preSMA-SMA ROI performed on rsfMRI data. Tables 7, 8, 9, 10, 11, and 12 show the same for the dMRI data.

The RSS, VMS, PSS, and USS refer to the memory usage during the execution of a task, with each metric providing a different measurement. Due to overlap between metrics it is not correct to sum them, instead they should be assessed individually. USS is likely the most representative metric for determining how much memory is actually being used by a process (Rodola, 2019). Note that the reported benchmarks are per run of a task, where each task ran a number of times equal to the *n jobs* column. For example, the connectivity task ran 300 times (once per subject), whereas the kmeans clustering task ran 1200 times (4 times per connectivity matrix, once for each requested value of k). Other metrics, such as I/O in, and I/O out were reported by Snakemake but not included here for the sake of brevity. All reported values are in megabytes (MB), except duration which is in the *hours:minutes:seconds* format. Since benchmarking is recorded in seconds, tasks that took less than half a second are rounded to 0 seconds.

Accurately testing runtime and CPU usage is difficult as it depends on many factors that can differ strongly between systems and configuration settings. For example, the size of the input data and ROI, as well as using local or shared computational resources may influence any of the reported metrics. With data sets as large as the HCP data, users will often be limited to using shared resources when processing this data with *CBPtools*. Total compute time for the entire procedure may therefore vary considerably even between runs on the same system. Thus, all reported benchmarks should only be interpreted as a loose guideline of what can be expected from this software.

3.2 Amygdala long-range dMRI connectivity

The dMRI clusterings of R amygdala reveal a mediolateral separation that splits further along the same medio-lateral axis at higher clustering granularity. Due to the strangeness of this layered pattern of clusterings as well as the problems inherent to probabilistic tractography and the poor signal to noise ratio of MRI in subcortical regions, we have further investigated the source of this clustering. To determine whether this pattern is driven solely by local connectivity rather than differentiation based on long-range connectivity profiles of the ROI voxels, the ROI and a border around it were excluded from the whole-brain gray matter target mask in a follow-up analysis. The exclusion of the ROI along with borders of 5, 20, and 40 mm around it were separately analyzed. Next, the ARI was computed between these clustering results and the original R amygdala clustering results (Fig. 5 and Fig. S6) to assess their similarity. Lastly, the target voxel contribution to the $k = 2$ clustering of the R amygdala was evaluated. The dMRI connectivity matrices, computed with PROBTRACKX2 as described in the paper (Sect. 2.1.4 Connectivity computation), were averaged for all subjects, resulting in an

Table 1. Duration per task for the rsfMRI preSMA-SMA parcellation

task	mean	std	min	max	n jobs
process masks	00:00:00	-	-	-	1
connectivity	00:07:51	00:02:47	00:02:55	00:13:25	300
kmeans clustering	00:03:07	00:00:52	00:01:25	00:05:14	1200
internal validity	00:00:06	00:00:00	00:00:05	00:00:07	300
group-level clustering	00:00:01	00:00:01	00:00:00	00:00:04	4
group similarity	00:00:07	-	-	-	1
plot individual similarity	00:00:02	00:00:00	00:00:01	00:00:02	4
plot group similarity	00:00:00	-	-	-	1
plot labeled ROI	00:00:04	00:00:00	00:00:03	00:00:04	24
plot internal validity	00:00:00	00:00:00	00:00:00	00:00:00	3

Table 2. Maximum resident set size (RSS) per task in MB for the rsfMRI preSMA-SMA parcellation

task	mean	std	min	max	n jobs
process masks	134.43	-	-	-	1
connectivity	468.99	637.46	205.34	3532.4	300
kmeans clustering	393.53	39.5986	318.15	464.35	1200
internal validity	298.86	11.713	283.92	373.33	300
group-level clustering	152.99	2.34687	149.63	155.21	4
group similarity	147.43	-	-	-	1
plot individual similarity	167.63	0.180278	167.5	167.94	4
plot group similarity	134.37	-	-	-	1
plot labeled ROI	158.79	0.244233	158.11	159.27	24
plot internal validity	137.63	2.22257	134.51	139.52	3

Table 3. Maximum virtual memory size (VMS) per task in MB for the rsfMRI preSMA-SMA parcellation

task	mean	std	min	max	n jobs
process masks	3191.87	-	-	-	1
connectivity	7465.29	36.2322	6958.66	7556.79	300
kmeans clustering	3644.03	38.031	3599.16	3676.32	1200
internal validity	3495.10	24.345	3459.93	3564.05	300
group-level clustering	3322.00	31.1755	3268	3340.02	4
group similarity	3338.63	-	-	-	1
plot individual similarity	3372.64	0	3372.64	3372.64	4
plot group similarity	3191.89	-	-	-	1
plot labeled ROI	3383.47	0.0128493	3383.45	3383.5	24
plot internal validity	3261.89	49.4904	3191.9	3296.89	3

Table 4. Maximum unique set size (USS) per task in MB for the rsfMRI preSMA-SMA parcellation

task	mean	std	min	max	n jobs
process masks	95.21	-	-	-	1
connectivity	427.68	642.234	162.85	3490.46	300
kmeans clustering	352.90	37.9777	276.42	418.92	1200
internal validity	257.04	11.8793	240.17	332.17	300
group-level clustering	109.41	2.91085	105.21	112.8	4
group similarity	102.34	-	-	-	1
plot individual similarity	124.50	1.74108	123.23	127.49	4
plot group similarity	90.58	-	-	-	1
plot labeled ROI	114.10	0.109287	113.89	114.25	24
plot internal validity	92.98	1.66349	90.64	94.36	3

Table 5. Maximum proportional set size (PSS) per task in MB for the rsfMRI preSMA-SMA parcellation

task	mean	std	min	max	n jobs
process masks	114.06	-	-	-	1
connectivity	433.62	642.058	173.45	3497.87	300
kmeans clustering	367.71	37.9632	291.49	434.04	1200
internal validity	272.84	11.7443	255.77	346.41	300
group-level clustering	120.80	4.83297	114.68	126.69	4
group similarity	110.40	-	-	-	1
plot individual similarity	140.05	6.08659	133.26	147	4
plot group similarity	98.17	-	-	-	1
plot labeled ROI	116.17	0.71444	115.42	117.78	24
plot internal validity	99.26	1.82353	96.69	100.73	3

Table 6. Maximum CPU load per second for each task in MB for the rsfMRI preSMA-SMA parcellation

task	mean	std	min	max	n jobs
process masks	0.00	-	-	-	1
connectivity	3.36	3.94097	0.54	21.85	300
kmeans clustering	260.10	48.1961	190.88	428.98	1200
internal validity	369.16	25.1319	300.23	449.27	300
group-level clustering	36.26	30.492	0	83.56	4
group similarity	92.30	-	-	-	1
plot individual similarity	111.55	2.65929	107.93	115.21	4
plot group similarity	0.00	-	-	-	1
plot labeled ROI	71.60	9.28152	57.32	86.26	24
plot internal validity	0.00	0	0	0	3

Table 7. Duration per task for the dMRI preSMA-SMA parcellation

task	mean	std	min	max	n jobs
process masks	00:00:00	-	-	-	1
connectivity	00:01:24	00:00:19	00:00:34	00:02:41	300
kmeans clustering	00:07:50	00:02:10	00:03:49	00:12:25	1200
internal validity	00:00:11	00:00:00	00:00:10	00:00:11	300
group-level clustering	00:00:01	00:00:01	00:00:00	00:00:04	4
group similarity	00:00:08	-	-	-	1
plot individual similarity	00:00:02	00:00:00	00:00:01	00:00:02	4
plot group similarity	00:00:00	-	-	-	1
plot labeled ROI	00:00:04	00:00:00	00:00:03	00:00:04	24
plot internal validity	00:00:00	00:00:00	00:00:00	00:00:00	3

Table 8. Maximum resident set size (RSS) per task in MB for the dMRI preSMA-SMA parcellation

task	mean	std	min	max	n jobs
process masks	134.91	-	-	-	1
connectivity	1151.82	108.917	859.34	1503.99	300
kmeans clustering	748.99	97.3975	547.84	915.24	1200
internal validity	468.60	31.9433	418.03	613.05	300
group-level clustering	151.87	2.41363	149.03	155.39	4
group similarity	146.80	-	-	-	1
plot individual similarity	164.48	5.83578	154.38	168.08	4
plot group similarity	134.84	-	-	-	1
plot labeled ROI	158.76	0.30115	158.21	159.38	24
plot internal validity	134.50	0.257207	134.14	134.7	3

Table 9. Maximum virtual memory size (VMS) per task in MB for the dMRI preSMA-SMA parcellation

task	mean	std	min	max	n jobs
process masks	3191.92	-	-	-	1
connectivity	4410.93	83.9623	4217.11	4737.08	300
kmeans clustering	4030.72	88.4106	3733.02	4098.15	1200
internal validity	3696.22	38.0761	3636.38	3829.43	300
group-level clustering	3304.01	36.005	3267.99	3340.02	4
group similarity	3338.65	-	-	-	1
plot individual similarity	3355.92	28.9974	3305.7	3372.68	4
plot group similarity	3191.91	-	-	-	1
plot labeled ROI	3383.48	0.0495798	3383.25	3383.51	24
plot internal validity	3191.90	0.00816497	3191.89	3191.91	3

Table 10. Maximum unique set size (USS) per task in MB for the dMRI preSMA-SMA parcellation

task	mean	std	min	max	n jobs
process masks	93.45	-	-	-	1
connectivity	1109.09	109.136	821.17	1463.93	300
kmeans clustering	707.34	97.206	504.75	869.72	1200
internal validity	425.04	31.7783	373.96	570	300
group-level clustering	108.36	1.9292	106.6	111.35	4
group similarity	103.77	-	-	-	1
plot individual similarity	121.44	5.85576	111.75	127.2	4
plot group similarity	91.03	-	-	-	1
plot labeled ROI	114.06	0.187705	113.59	114.66	24
plot internal validity	90.72	0.139603	90.52	90.83	3

Table 11. Maximum proportional set size (PSS) per task in MB for the dMRI preSMA-SMA parcellation

task	mean	std	min	max	n jobs
process masks	113.51	-	-	-	1
connectivity	1128.20	109.084	839.71	1482.42	300
kmeans clustering	723.25	97.2084	519.75	883.56	1200
internal validity	440.07	31.6481	391.82	585.34	300
group-level clustering	118.84	2.76936	115.67	122.74	4
group similarity	111.25	-	-	-	1
plot individual similarity	135.31	9.35706	120.77	146.83	4
plot group similarity	99.00	-	-	-	1
plot labeled ROI	116.08	0.71373	115.54	118.66	24
plot internal validity	97.63	0.417692	97.04	97.95	3

Table 12. Maximum CPU load per second for each task in MB for the dMRI preSMA-SMA parcellation

task	mean	std	min	max	n jobs
process masks	0.00	-	-	-	1
connectivity	98.11	1.71986	85.59	101.79	300
kmeans clustering	208.21	32.7676	162.54	295.77	1200
internal validity	365.97	13.5011	343.32	418.99	300
group-level clustering	32.65	35.3829	0	84.58	4
group similarity	88.92	-	-	-	1
plot individual similarity	91.03	52.8042	0	128.09	4
plot group similarity	0.00	-	-	-	1
plot labeled ROI	68.86	8.37796	49.62	80.61	24
plot internal validity	0.00	0	0	0	3

ROI voxel by target voxel matrix. The ROI voxels were separated by cluster, and the connectivity values of each target voxel to all ROI voxels within a cluster were then averaged. This provided an average connectivity value of each target voxel to each of the two clusters. The Euclidean distance between the connectivity values of both clusters for each target voxel was then calculated. The higher this distance, the larger of a contribution this target voxel provided to separating both clusters. This array of Euclidean distance values per target voxel was then z-scored and values below 1.96 were removed. The remaining target voxels were considered to be the most important features as per their contribution to the cluster separation for $k = 2$.

While the visual representations of the clusters mapped onto the R amygdala ROI (Fig. S7) look relatively similar, the ARI scores reveal that this is not the case as more local connections are removed from the target features (Fig. S8). The $k = 2$ cluster solution for the 5mm run had an ARI of .9, whereas the 20 mm and 40 mm solutions had .8 and .46, respectively. At higher clustering granularities, the 40 mm solution remained at low similarity to the original clustering solution. However, while the 5 mm and 20 mm results each revealed less similarity at the $k = 3$ granularity, they became more similar to the original solution again at $k = 4$ and 5. Nonetheless, two of the three assessed validity indices, the Silhouette index and the Calinski-Harabasz index, indicated that the 2-cluster solution best fitted the data as per the original clustering results, as well as the 5 mm, 20 mm, and 40 mm results. The Davies-Bouldin index suggested a best fitting 3-cluster solution for the original results as well as the 5 mm results. For the 20 mm and 40 mm results, however, the Davies-Bouldin index instead suggested a better fitting 5-cluster solution. Taken together with a decrease in similarity as a larger area of local connectivity was excluded from the target features, this may hint that a more strongly coherent 2- or 3-cluster solution was driven predominantly by patterns in local connectivity.

Fig. S9 is a visual representation of the target voxels that most contributed to the separation of clusters of the original 2-cluster solution. These voxels are mostly in the same hemisphere as the ROI, with a handful of voxels located in the left hemispheric amygdala. However, more interhemispheric connections may be expected to the left amygdala, as the medial amygdala is suggested to be strongly connected with its interhemispheric counterpart. Only a handful of voxels being present here may have been caused by lower sensitivity in probabilistic tractography on account of the thresholds used to improve specificity and counteract false positives.

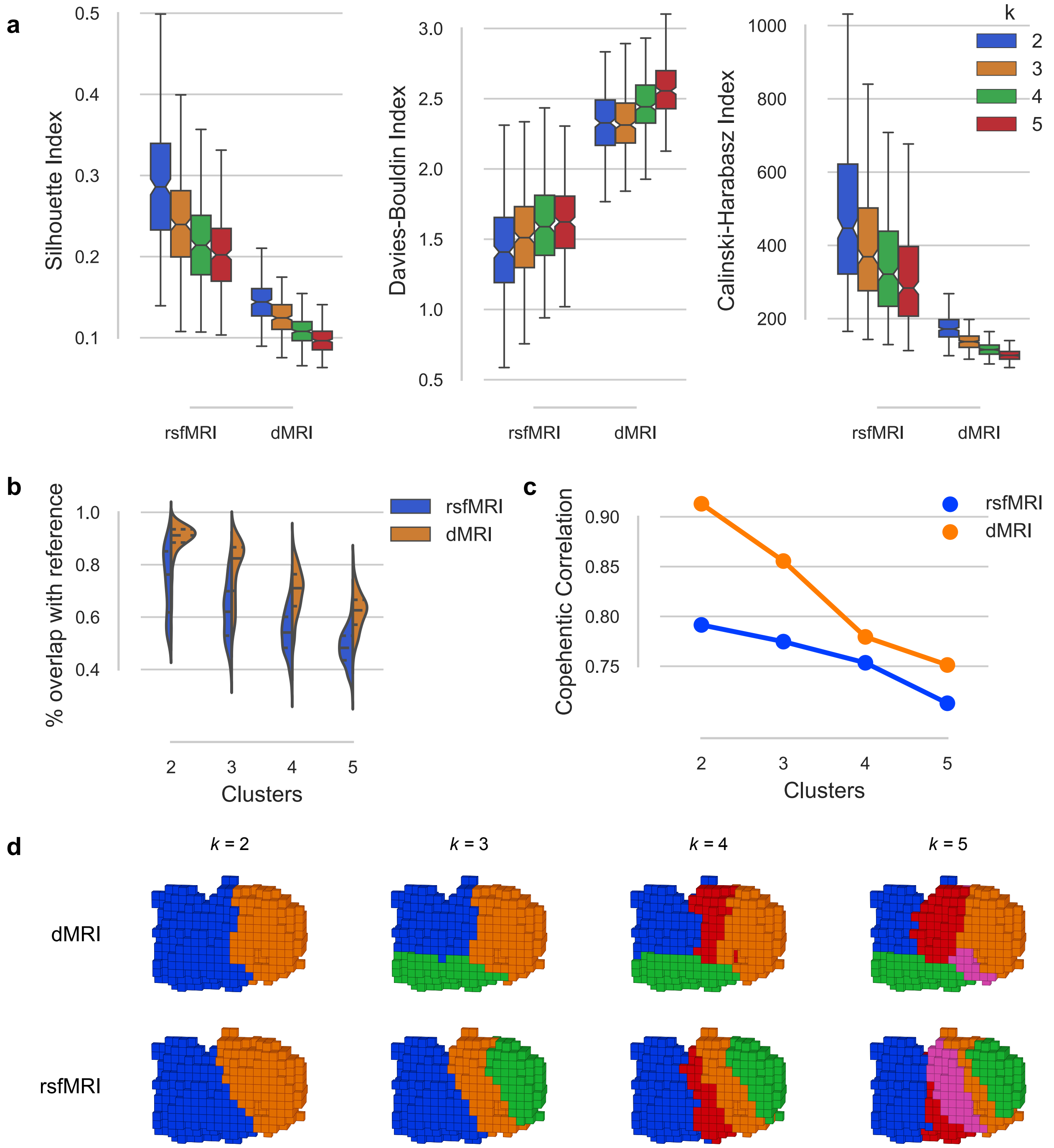

Fig. S4 R preSMA-SMA validity metrics and parcels. **a** Internal validity scores (the Silhouette index, the Davies-Bouldin index, and the Calinski-Harabasz index) for all tested solutions ($k = [2, 3, 4, 5]$). **b** Accuracy of the relabeling for each individual clustering to the group-clustering, with the cluster number k on the x-axis, comparing rsfMRI (blue) to dMRI (orange). **c** Cophenetic correlation scores of the reference clusterings for $k = [2, 3, 4, 5]$ based on the rsfMRI (blue) and dMRI (orange) modalities. **d** Parcels for the $k = [2, 3, 4, 5]$ cluster solutions for both dMRI (top row) and rsfMRI (bottom row). The view is from the right side of the ROI with the posterior on the left and anterior on the right (the same view as applied in Fig. 2B of the paper)

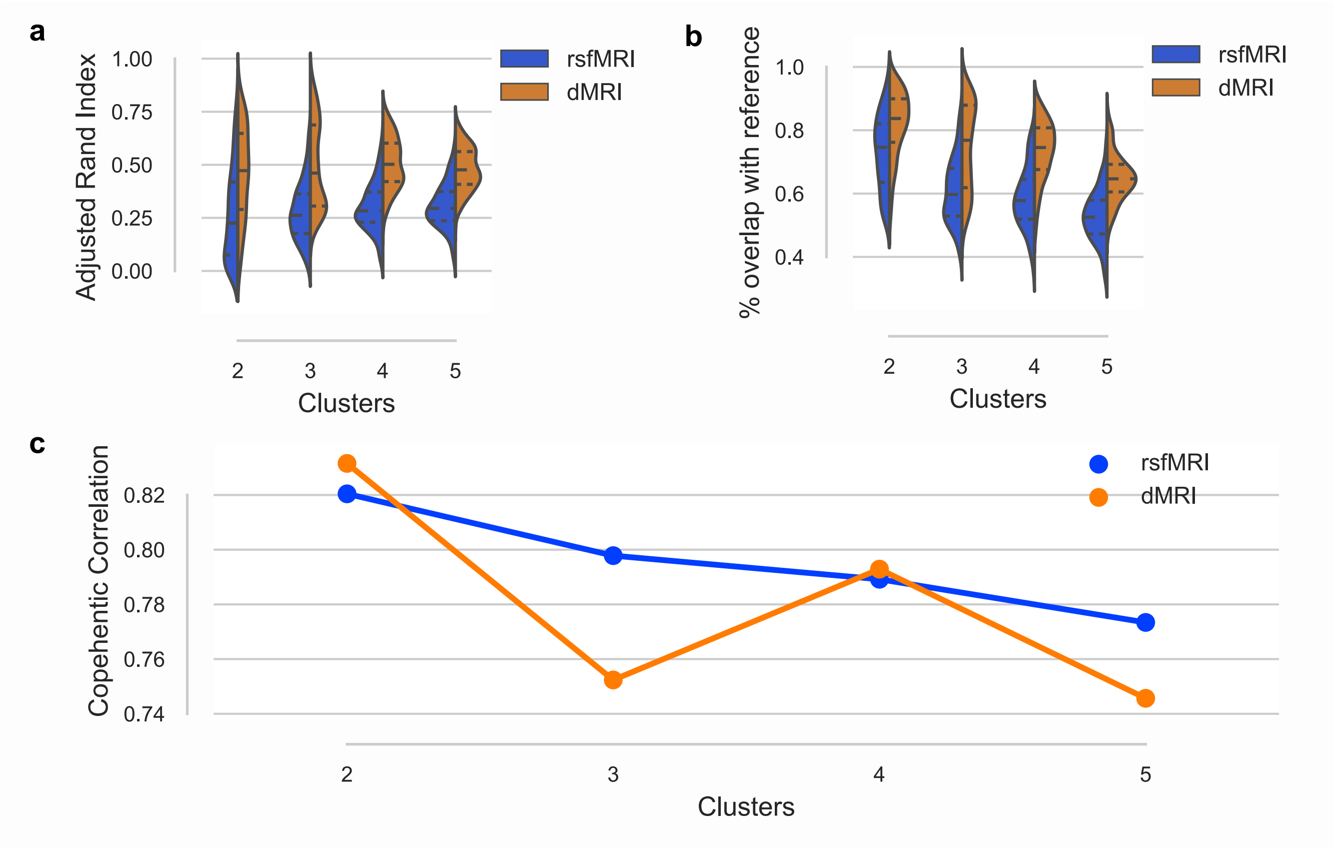

Fig. S5 Validity metrics for the R insula. **a** Group similarity scores (i.e., the similarity of individual clusterings to the group-clustering) using the ARI, with the cluster number k on the x-axis, comparing rsfMRI (blue) to dMRI (orange). **b** Accuracy of the relabeling of individual subject cluster labels to a reference clustering, calculated as the percentage of overlap between both clusterings. **c** Cophenetic correlation scores of the reference clustering for $k = [2, 3, 4, 5]$ based on the rsfMRI (blue) and dMRI (orange) modalities

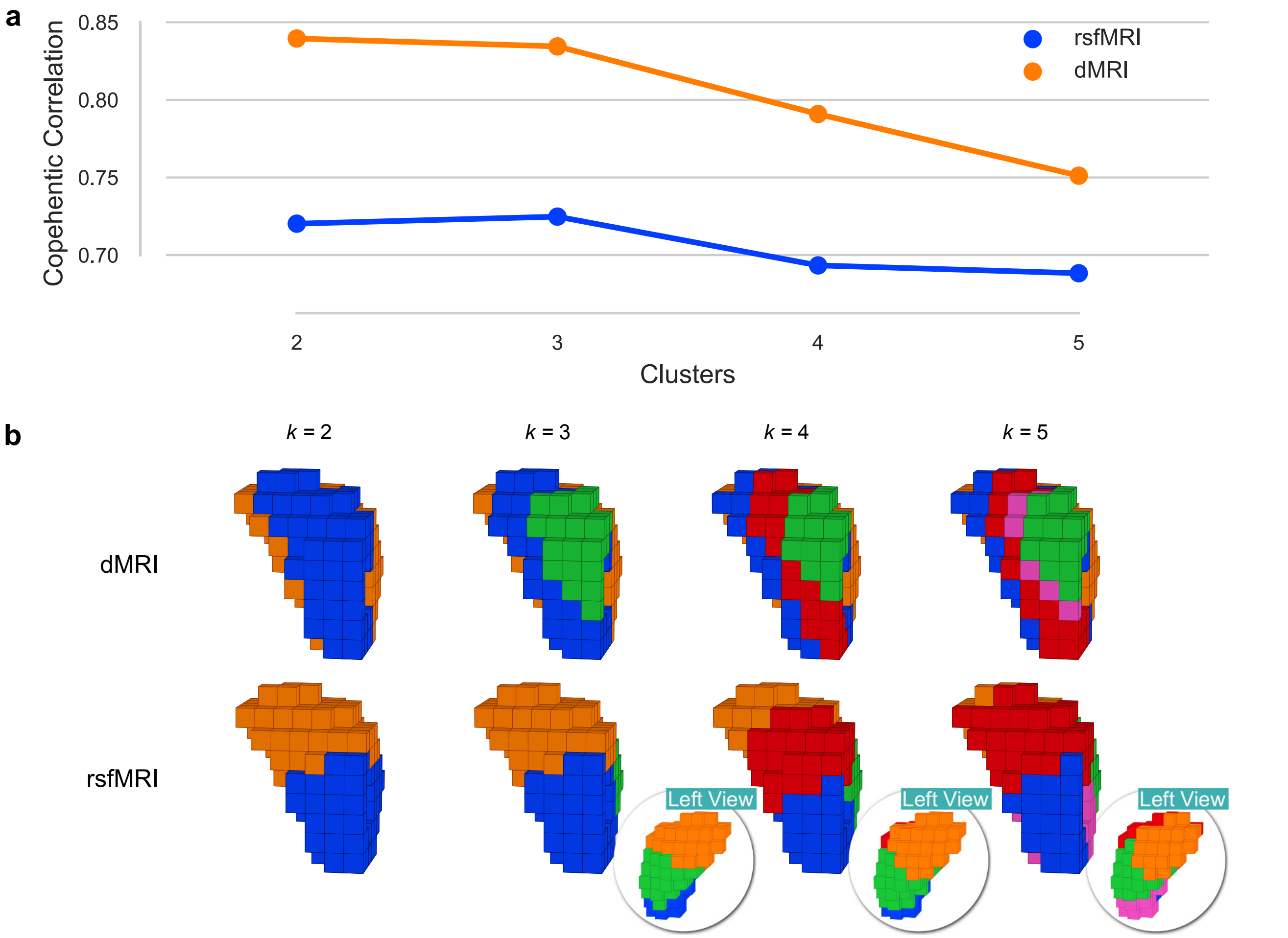

Fig. S6 R amygdala metrics and parcels for the 2, 3, 4, and 5-cluster solutions. **a** Cophenetic correlation scores of the reference clustering for both rsfMRI (blue) and dMRI (orange). **b** Cluster solutions for all investigated values of k mapped onto the original ROI image, for both dMRI (top row) and rsfMRI (bottom row)

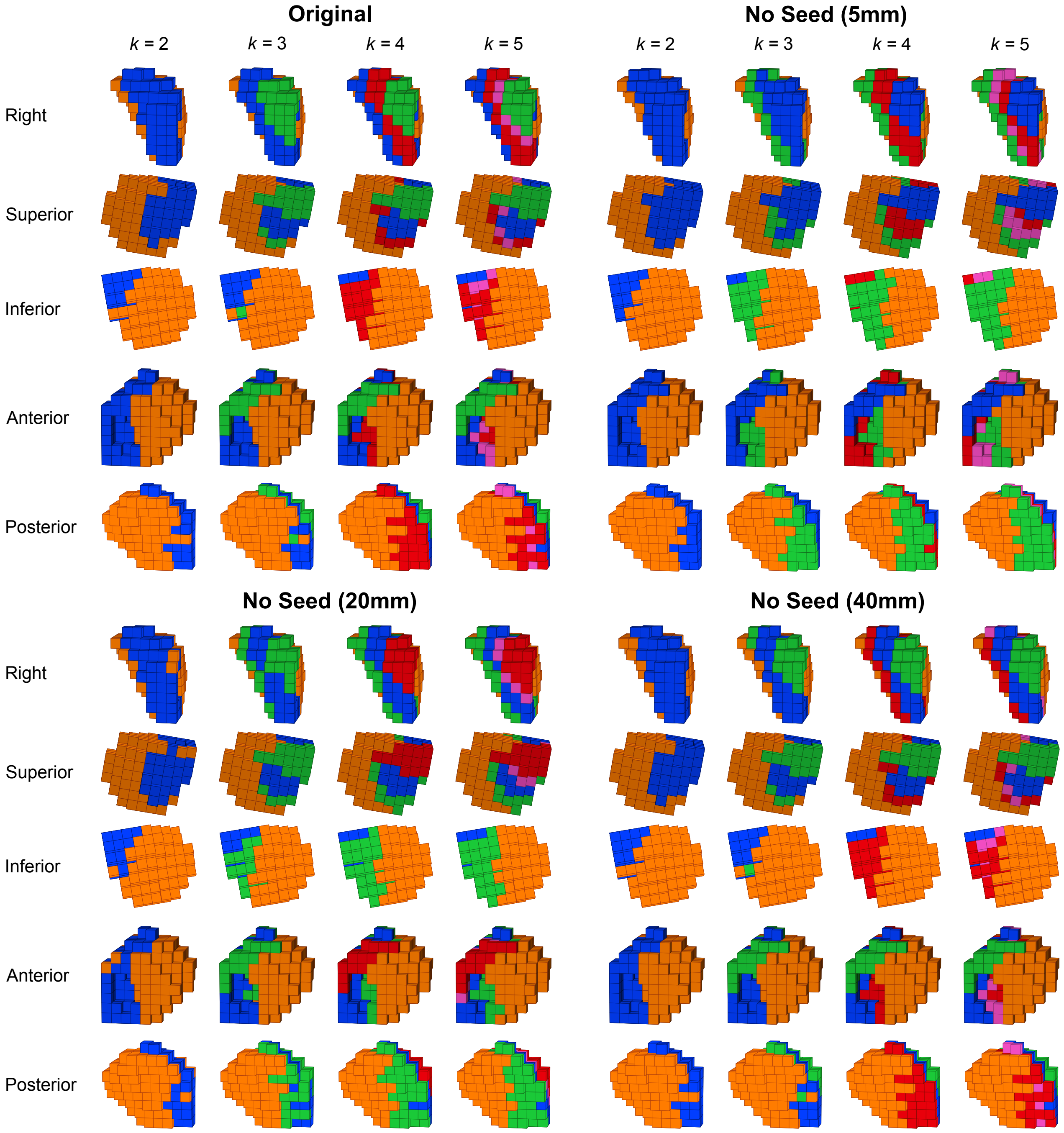

Fig. S7 R amygdala parcels for $k = [2, 3, 4, 5]$ using different target features. The top left shows the parcels when ROI voxels have a connectivity profile containing all whole-brain gray matter voxels. The top right shows the parcels that result from extracting the ROI voxels from the target features including a border of 5 mm around the ROI. The bottom left and right show the same, but with a border of 20 and 40 mm respectively

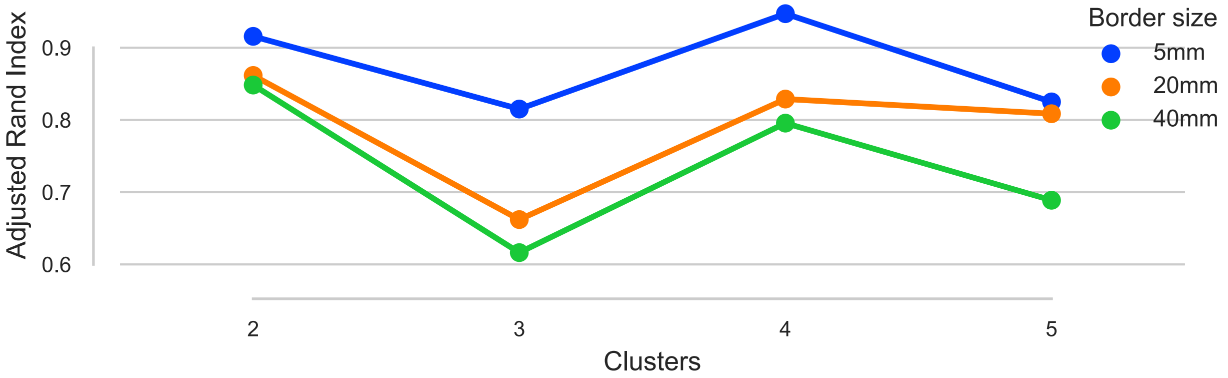

Fig. S8 Similarity between R amygdala clustering results where local connectivity was excluded from the target features (Fig. S7) and the clustering results where all whole-brain gray matter voxels were used as target features (Fig. 5 and Fig. S6), for $k = [2, 3, 4, 5]$

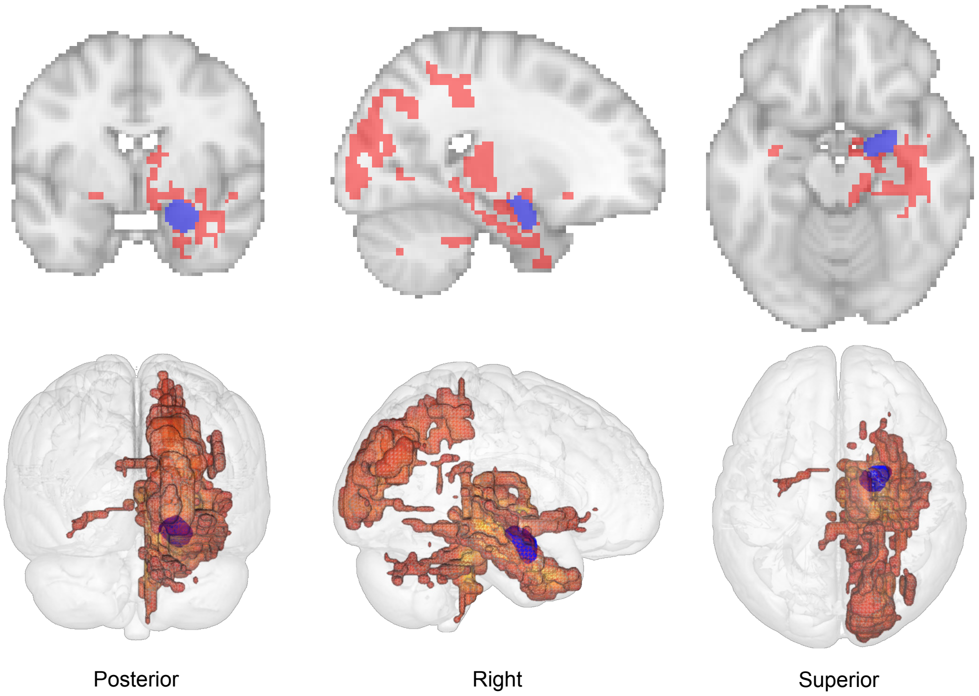

Fig. S9 Mapping of target voxels (red) that most contribute to the $k = 2$ cluster formation of the R amygdala (blue). The top row of figures was generated using Nilearn's plotting tools (Abraham et al., 2014), whereas the bottom row was generated using Mango (multi-image analysis GUI; http://ric.uthscsa.edu/mango/)

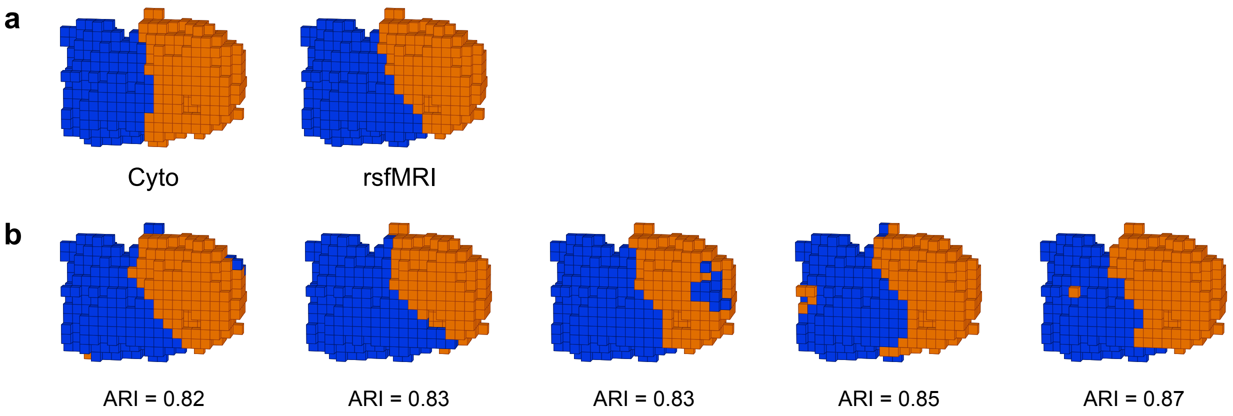

Fig. S10 Individual subject clustering solutions for the rsfMRI 2-cluster solution of the R preSMA and SMA ROI. **a** The 2-cluster solutions of the combined R preSMA and SMA ROI for the cytoarchitectonically defined (Ruan et al., 2018) subdivision from the Jülich histological atlas (Eickhoff et al., 2005), and the rsfMRI connectivity-based group-level parcels. **b** Subject-level clusterings of five subjects for the rsfMRI preSMA-SMA 2-cluster solution and their adjusted Rand index (ARI) values to the rsfMRI group-level parcellation

References

Abraham A, Pedregosa F, Eickenberg M, Gervais P, Mueller A, Kossaifi J, Gramfort A, Thirion B, Varoquaux G (2014) Machine learning for neuroimaging with scikit-learn. Frontiers in Neuroinformatics 8:14, DOI 10.3389/fninf.2014.00014

Eickhoff SB, Stephan KE, Mohlberg H, Grefkes C, Fink GR, Amunts K, Zilles K (2005) A new SPM toolbox for combining probabilistic cytoarchitectonic maps and functional imaging data. Neuroimage 25(4):1325–1335, DOI 10.1016/j.neuroimage.2004.12.034

Halchenko YO, Hanke M, Poldrack B, Meyer K, Solanky DS, Alteva G, Gors J, MacFarlane D, Olaf Häusler C, Olson T, et al (2019a) datalad/datalad 0.11.5. Zenodo DOI 10.5281/zenodo.3233911

Halchenko YO, Hanke M, Poldrack B, Meyer K, Solanky DS, Alteva G, Gors J, MacFarlane D, Olaf Häusler C, Olson T, et al (2019b) datalad/datalad 0.12.0rc6. Zenodo DOI 10.5281/zenodo.3512712

Köster J (2019) Snakemake documentation. URL https://snakemake.readthedocs.io/en/stable/, accessed: 18 Dec 2019

Köster J, Rahmann S (2012) Snakemake–a scalable bioinformatics workflow engine. Bioinformatics 28(19):2520–2522, DOI 10.1093/bioinformatics/bts480

Nguyen N, Caruana R (2007) Consensus Clusterings, IEEE, p 607–612. DOI 10.1109/ICDM.2007.73

Pedregosa F, Varoquaux G, Gramfort A, Michel V, Thirion B, Grisel O, Blondel M, Prettenhofer P, Weiss R, Dubourg V, Vanderplas J, Passos A, Cournapeau D, Brucher M, Perrot M, Duchesnay É (2011) Scikit-learn: Machine learning in Python. Journal of Machine Learning Research URL http://jmlr.csail.mit.edu/papers/v12/pedregosa11a.html

Reuter N (2019) CBPtools documentation. URL https://cbptools.readthedocs.io/en/latest/, accessed: 18 Dec 2019

Rodola G (2019) psutil documentation. URL https://psutil.readthedocs.io/en/latest/, accessed: 18 Dec 2019

Ruan J, Bludau S, Palomero-Gallagher N, Caspers S, Mohlberg H, Eickhoff SB, Seitz RJ, Amunts K (2018) Cytoarchitecture, probability maps, and functions of the human supplementary and pre-supplementary motor areas. Brain Struct Funct 223(9):4169–4186, DOI 10.1007/s00429-018-1738-6
